# Probiotic colonization of *Xenopus laevis* skin causes short-term changes in skin microbiomes and gene expression

**DOI:** 10.1128/iai.00569-24

**Published:** 2025-04-02

**Authors:** Joseph D. Madison, Owen G. Osborne, Amy Ellison, Christina N. Garvey Griffith, Lindsey Gentry, Harald Gross, Brian Gratwicke, Leon Grayfer, Carly R. Muletz-Wolz

**Affiliations:** 1 Center for Conservation Genomics, Smithsonian’s National Zoo and Conservation Biology Institute 189732 , Washington, DC, USA; 2 Department of Biology, University of Massachusetts Boston 168394, Boston, Massachusetts, USA; 3 Department of Biology, Xavier University of Louisiana 542200, New Orleans, Louisiana, USA; 4 School of Environmental and Natural Sciences, Bangor University 1506, Bangor, United Kingdom; 5 Department of Biology, George Washington University 8367, Washington, DC, USA; 6 Department of Pharmaceutical Biology, Pharmaceutical Institute, University of Tübingen 9188, Tübingen, Germany; 7 Center for Species Survival, Smithsonian’s National Zoo and Conservation Biology Institute, Washington, DC, USA; University of California San Diego School of Medicine, La Jolla, California, USA

**Keywords:** microbiome, amphibian, immune response, probiotics, microbial ecology, disease ecology, transcriptomics, metagenomics

## Abstract

Probiotic therapies have been suggested for amelioration efforts of wildlife disease such as chytridiomycosis caused by *Batrachochytrium* spp. in amphibians. However, there is a lack of information on how probiotic application affects resident microbial communities and immune responses. To better understand these interactions, we hypothesized that probiotic application would alter microbial community composition and host immune expression in *Xenopus laevis*. Accordingly, we applied three amphibian-derived and anti-*Batrachochytrium* bacteria strains (two *Pseudomonas* spp. and one *Stenotrophomonas* sp.) to *X. laevis* in monoculture and also as a cocktail. We quantified microbial community structure using 16S rRNA gene sequencing. We also quantified genes involved in *X. laevis* immune responses using quantitative reverse transcriptase polymerase chain reaction (RT-qPCR) and skin transcriptomics over 1 and 3-week periods. All probiotic treatments successfully colonized *X. laevis* skin for 3 weeks, but with differential amplicon sequence variant (ASV) sequence counts over time. Bacterial community and immune gene effects were most pronounced at week 1 post-probiotic exposure and decreased thereafter. All probiotic treatments caused initial changes to bacterial community alpha and beta diversity, including reduction in diversity from pre-exposure anti-*Batrachochytrium* bacterial ASV relative abundance. Probiotic colonization by *Pseudomonas* probiotic strain RSB5.4 reduced expression of regulatory T cell marker (*FOXP3,* measured with RT-qPCR) and caused the greatest gene expression changes detected by transcriptomics. Single bacterial strains and mixed cultures, therefore, altered amphibian microbiome-immune interactions. This work will help to improve our understanding of the role of the microbiome-immune interface underlying both disease dynamics and emergent eco-evolutionary processes.

**IMPORTANCE:**

Amphibian skin microbial communities have an important role in determining disease outcomes, in part through complex yet poorly understood interactions with host immune systems. Here we report that probiotic-induced changes to the *Xenopus laevis* frog skin microbial communities also result in significant alterations to these animals’ immune gene expression. These findings underscore the interdependence of amphibian skin immune-microbiome interactions.

## INTRODUCTION

Probiotics are widely being suggested as treatment options for a variety of diseases in wildlife, agriculture, and human health (1, 2), albeit with caveats and considerations for their applicability (3, 4). Yet, the impact of adding probiotic bacteria on host-associated microbial communities and host immune responses remains an understudied frontier in the microbial sciences (5, 6). Explicating these combinative microbial-immune effects has important implications in understanding disease emergence, host-microbial ecology, and related evolutionary trends (7–9).

Of the various metazoan-microbe systems under study, amphibian-microbiome-disease systems are of exceptional utility for addressing these questions due to their ease of manipulation in the laboratory, extensive baseline data on the skin microbiota of various species, and applicability to disease-mediated population declines (10, 11). For disease questions, the causative fungal pathogens of amphibian chytridiomycosis, *Batrachochytrium dendrobatidis* (*Bd*) and *Batrachochytrium salamandrivorans* (*Bsal*), are of widespread interest (12–14). However, there remain important questions that are unaddressed in these systems. Specifically, the mechanisms underpinning cutaneous probiotic modulation on host innate and adaptive immune responses in amphibians remain poorly understood (15).

Understanding if probiotics modulate the immune system and promote microbial community restructuring has important implications for disease amelioration efforts and the basic understanding of immune-microbiome interaction in metazoan disease ecology and evolution. Previous work with probiotics or microbiome manipulations in amphibians has shown differential effects on host immune responses. For example, application of known anti-*Bd* probiotics in *Rana sierrae* resulted in downregulation of defensive skin anti-microbial peptides (AMPs) and altered microbial community composition (16), indicating a potential for probiotic-based AMP regulation. In *Plethodon cinereus*, temperature treatments caused shifts in the skin microbiome (17), and impacted immune gene expression profiles and disease outcome (18). Specifically, lower temperatures and *Bd* exposure were linked to decreasing bacterial richness, increased inflammation, and higher *Bd* loads. Yet, beyond these examples, there are few studies examining the microbial-immune interface in amphibians and in vertebrates more broadly (1, 19).

Previous work with single bacteria strain exposures has indicated varied microbial community responses in a disease context. In amphibians, some of these experiments reported beneficial effects for amphibian hosts against *Bd* infection (20, 21), while others indicated limited or no significant effects (22–25). Results with variability in beneficial effects have also been seen in probiotic inoculation of bats to modulate disease outcomes from the fungal pathogen *Pseudogymnoascus destructans* (26). Our inability to successfully alter microbiomes stems, at least in part, from a poor understanding of the microbial-immune interface that is not unique to amphibian systems. In turn, identifying the mechanisms of microbiome-immune cross-talk will increase our ability to make applied use of microbiomes (27). Here, we hypothesized that probiotic exposure with three previously identified anti-*Bd* bacteria (28) would result in skin colonization of the model frog species, *Xenopus laevis*, and that these bacteria would persist after exposure ceased as observed in other amphibian systems (23, 25). Second, we hypothesized that probiotic exposure in *X. laevis* would alter both the resident microbiome and the expression profiles of key regulatory genes involved in the *X. laevis* innate and adaptive immune response.

The results of this work show that all probiotics applied in our experiment can persist for at least 3 weeks and cause shifts in the microbial community and immune gene expression. Notably, we identified conserved immune changes caused by all probiotic treatments. This includes multiple genes identified through transcriptomics that indicate both T cell regulation/differentiation and also activation of the *X. laevis* complement cascade. Moreover, we found that one *Pseudomonas* probiotic caused downregulation of expression of transcription factor forkhead box P3 (FOXP3), a conserved marker of regulatory T cells (Tregs; 29) and caused the largest shifts to host immune gene expression compared to other probiotic treatments. We thus document an important association between amphibian skin-associated bacteria and immune responses, giving impetus for future research directions examining constraints on the amphibian adaptive response by their microbial communities.

## MATERIALS AND METHODS

### Probiotic selection and genome sequencing

We used three bacterial strains and a combination of the three strains as probiotic treatments for this study. These strains were selected from a collection of 119 bacterial isolates that were previously isolated from *Plethodon* salamanders in Maryland and Virginia, USA (28). The three strains were selected because they had >90% inhibition strength against *Bd* strain JEL404 (28), and also matched at 100% sequence similarity to bacterial amplicon sequence variants (ASVs) detected in DNA skin microbiome samples from multiple amphibian species including: Appalachian salamanders (30, 31), the Panamanian golden frog *Atelopus zeteki* (25), and *Xenopus laevis* (32). Each of the three strains was sequenced on Oxford Nanopore GridION machines. Their genomes were assembled using Flye 2.9 (33) and polished with Medaka 1.4.3. We used the web-based tools Type Strain Genome Server (TYGS) (34) and autoMLST (35) for automated whole genome-based analyses in order to determine their taxonomic identity. *Pseudomonas* RSB5.4 (Probiotic 1 [P1], ASV18) was isolated from *Plethodon cinereus* in Shenandoah National Park (NP), Virginia, and also has been shown to be inhibitory against multiple *Bd* strains and *Bsal* across temperatures (36). Genome-based taxonomic analyses employing TYGS revealed that *Pseudomonas koreensis* LMG 21318^T^ represents the closest related type strain of RSB5.4. In pairwise comparisons, independent of the applied Genome BLAST Distance Phylogeny formula, a digital DNA-DNA hybridization (dDDH) value *d*_4_ of 43.4% was calculated. Since these values are well below the species threshold of 70%, strain RSB 5.4 possibly represents a new candidate *Pseudomonas* species. This tentative finding was complemented by an analysis of the average nucleotide identity (ANI) using autoMLST, which identified *Pseudomonas granadensis*^T^ with 89.4% ANI as the most similar type strain. Since this value is also well below the one for species delineation and since values between 88% and 90% indicate a new subspecies (37), the ANI-based analysis also supported the TYGS results, and RSB 5.4 truly represents a new subspecies. *Stenotrophomonas* THA2.2 (Probiotic 2 [P2], ASV9) was isolated from *Plethodon cylindraceus* in Shenandoah NP and was found to be a common bacterial symbiont of *Xenopus laevis* (32) and is, according to ANI analysis, most closely related to *Stenotrophomonas rhizophilia* DSM 14405^T^. Between THA 2.2 and DSM 14405^T^, an ANI value of 96.6% was determined, which clearly classifies THA2.2 as *S. rhizophila. Pseudomonas tolaasii* RSB5.11 (Probiotic 3 [P3], ASV10) was isolated from the same *P. cinereus* as RSB5.4. RSB5.11 was unambiguously identified as *P. tolaasii* given a dDDH-*d*_4_ value of 94.2% in pairwise comparison with *P. tolaasii* NCPPB 2192^T^. RSB5.11 has also been found to produce pseudodesmin A; a lipodepsipeptide shown to have inhibitory action against *Bd* and *Bsal* (38). Our final probiotic treatment was a cocktail of the three previous probiotics (probiotic 4: P4).

### Bacterial growth and inoculation

Each bacterial isolate was removed from long-term cryopreservation and passaged in 1% tryptone broth. To prepare the bacteria for use in the experiment, 5 mL of each bacterial strain was taken from a stock and added to 150 mL of 1% tryptone broth, followed by growth at 17°C for 68 h. The bacteria were then washed twice by centrifugation (4,500 rpm, 10 min) to remove detrimental metabolites and re-suspended in sterilized reverse osmosis (RO) water (21). Bacteria used to inoculate in the experiment were kept at 4°C until used each day.

### Animal sourcing and husbandry

Outbred 1-year-old (1.5″–2″), mixed sex *X. laevis* were purchased from Xenopus 1 (Dexter, MI). All animals were housed and handled under strict laboratory regulations as per GWU IACUC (approval number 15-024). For experimental probiotic application, animals were housed in individual 6″ × 6″ containers. During the course of the experiment, animals were kept on a 12 h day-night cycle in individual aquaria containing 200 mL of water and fed bi-weekly.

### Experimental design

The experimental setup entailed a 5 × 1 design, with the five treatment groups consisting of four experimental groups and one control group (Table 1). The experiment lasted 22 days. Each treatment group started the experiment with 18 individuals, with the experiment having a total of 90 individuals. Based on prior experiments (21, 25), we used 1 mL from a 6 × 10^7^ cells/mL bacterial stock of either each probiotic in monoculture (*Pseudomonas* RSB5.4 [P1], *Stenotrophomonas* THA2.2 [P2], *Pseudomonas tolaasii* RSB5.11 [P3]) or the probiotics combined as a cocktail (P4) to inoculate an aquarium containing 200 mL of water. This resulted in an exposure of 300,000 cells/mL for probiotic-treated frogs in the aquaria. Control (C) aquaria were inoculated with 1 mL of sterilized RO water. Frogs were then inoculated with their respective probiotics (experimental groups) or sterilized RO water (control group) on days 0, 2, 4, and 6 as per previous work (25). On day 6, all frogs remained treated in the probiotic solution (or water for controls) for 8 h. Then after 8 h of final treatment exposure on day 6, all aquaria water was discarded, and new water was added.

**TABLE 1:** Number of *X. laevis* used by treatment group (*Pseudomonas* RSB5.4 [P1], *Stenotrophomonas* THA2.2 [P2], *Pseudomonas tolaasii* RSB5.11 [P3], and cocktail [P4]), time point (initial cohort size, week 1, week 2, and week 3), and sample type (microbiome [M] or tissue [T])^*a*^

	RSB5.4 (P1)	THA2.2 (P2)	RSB5.11 (P3)	Cocktail (P4)	Controls
Initial cohort size	M = 18 (16)	M = 18 (15)	M = 18 (16)	M = 18 (17)	M = 18 (16)
Week 1	M = 18 (16); T = 12	M = 18 (17); T = 12	M = 18 (18); T = 12	M = 18 (18); T = 12	M = 18 (18); T = 12
Week 2	M = 6 (5)	M = 6 (6)	M = 6 (6)	M = 6 (6)	M = 6 (6)
Week 3	M = 6 (6); T = 6	M = 6 (6); T = 6	M = 6 (6); T = 6	M = 6 (6); T = 6	M = 6 (6); T = 6

^*a*^ A total of 90 *X*. *laevis* were used for the experiment. The number of individuals included in statistical analysis after data preprocessing is given in parentheses.

At specific time points, individual frogs were swabbed for microbiome quantification (days 0, 8, 15, and 22) and tissues were collected for quantitative reverse transcriptase polymerase chain reaction (RT-qPCR) (days 8 and 22). At days 0 (day 0) and 8 (week 1), all frogs were swabbed with 20 strokes on their entire ventral side after being rinsed with sterile RO water. This method follows previous studies using a similar swabbing procedure (39, 40). After swabbing on day 8 (week 1), 12 individuals per treatment were humanely euthanized for tissue collection. For tissue collection, a large piece of skin was excised with RNase AWAY (ThermoFisher, Waltham, MA) sterilized scissors and put in RNAlater (ThermoFisher, Waltham, MA), stored at 4°C for 24 h and then at −20°C until RNA extraction was performed. At day 15 (week 2) and 22 (week 3), all remaining frogs (*n* = 6/treatment) were swabbed. After swabbing at day 22 (week 3), the remaining individuals (*n* = 6/treatment) were humanely euthanized for tissue collection.

### Nucleic acid isolation

Microbiome swabs had DNA extracted using a Qiagen DNeasy PowerSoil Pro kit (Qiagen, Germantown, MD). Minor modifications to the manufacturer’s protocol included incubation of the swab in C1 buffer at 65°C for 10 min at 40 rpm, bead-beating for 90 s on a Mini-Beadbeater-96 (Biospec, Bartlesville, OK), and prior to the final elution having C6 warmed to 60°C and added to samples to incubate for 5 min.

### 16S rRNA gene sequencing

Extracted DNA from samples, positive microbiome controls (Zymo Research, Irvine, CA, USA: Cat No: 6300, 6305), and negative controls (extraction and PCR controls) were prepared for 16S rRNA gene sequencing using barcoded 515F-Y and 939R (V3–V5 region) primers and sequenced on an Illumina MiSeq with v3 2 × 300 chemistry following methods as fully described in Bornbusch et al. (41).

### RT-qPCR

RT-qPCR was used to quantify gene expression profiles of eight genes (Table 2) involved in immune system function. Skin tissues were homogenized in TRIzol reagent (ThermoFisher, Waltham, MA), flash frozen on dry ice, and stored at −80°C until RNA isolation. RNA isolation was performed using TRIzol according to the manufacturer’s directions. For RT-qPCR gene expression analysis, RNA (500 ng/sample) was reverse transcribed into cDNA using qScript cDNA supermix (QuantaBio, Beverly, MA).

**TABLE 2:** List of primer sequences for RT-qPCR

Gene name	Primer sequences (5′→3′; Fwd, Rev)
*FOXP3*	(F)-ATGGCACGGTTGTCTGGAGA (R)-CAAGCTGTTCTTCTAGTTTGTG
*IL10*	(F)-CAGTCCGTGTCTGAAACAATTC (R)-CAGCAACTTGTCCTTGAGAAAG
*CSF1*	(F)-GCCTCATATCATGCATCGTGGGAA (R)-TGTGTTCCGTGAAGCTGTCTCCTA
*IL34*	(F)-TGATAAGCGATTGACCTACCTGGG (R)-AGCTCTTCTACGGTGATTCCTTGG
*TNFA*	(F)-TGTCAGGCAGGAAAGAAGCA (R)-CAGCAGAGCAAAGAGGATGGT
*TLR2*	(F)-GCCATGGAGAAGAGCTACAA (R)-CAAAGAGACGGAAGTGAGAGAA
*TLR6*	(F)-CAGTCAGGAAGACTCAGAATGG (R)-CAATGATTGCTTTGCCAGGAATA
*TGFB*	(F)-CCTTACATCTGGAGCACAGATAC (R)-GGAACACAGCAGGGAGAAAT
*GAPDH*	(F)-ATGTGTCCGTTGTGGATCTG (R)-GATTCCTTTCATTGGTCCCTCT

Quantitative gene expression analysis on cDNA was performed using the CFX96 Real-Time System (Bio-Rad Laboratories, Hercules, CA) and iTaq Universal SYBR Green Supermix (Bio-Rad Laboratories). The Bio-Rad CFX Manager software (SDS) was employed for initial gene expression data capture. All downstream expression analyses were conducted using the ΔΔCt method relative to the *GAPDH* endogenous control gene for *X. laevis* (42). The primers used are listed in Table 2.

### Transcriptomics

Skin samples from weeks 1 and 3 timepoints were used for transcriptome sequencing. Total RNA was purified with the EZ2 Connect instrument (Qiagen) using the EZ2 RNA/miRNA Tissue/Cells Kit (Qiagen). RNA extracts were sent to Azenta Life Sciences for rRNA depletion, library construction, and sequencing. Dual depletion of bacterial and animal rRNA was conducted using the NEBNext rRNA (bacteria and human/mouse/rat) Depletion Kits (New England Biolabs), respectively. TruSeq 2 × 150 bp libraries were prepared and sequenced on a NovaSeq sequencing instrument (Illumina) to produce ~40 M read pairs per sample.

### Data analysis and statistics: 16S rRNA gene sequencing

Raw sequencing data in fastq.gz format was first preprocessed with dada2 (43, Jupyter Notebook, dada2). This was followed by an additional cleanup step for the removal of contaminants with the decontam package (44) and also a manual inspection of raw data for contaminants, subject to removal where appropriate (13 ASVs composing ~1% of taxa identified and removed as contaminants; Jupyter Notebook, Preprocessing). Singletons and non-target organelle reads from chloroplasts were also removed during preprocessing. These cleanup steps resulted in a total of 3,840,280 reads (2,676 unique taxa and 262 samples) being reduced to 3,502,980 reads (1,190 unique taxa and 240 samples; Jupyter Notebook, Preprocessing). All remaining samples were then rarefied to an even depth of 2,575 reads/sample using the rarefy_even_depth function in phyloseq and following results from rarefaction richness curve analysis (Fig. S1). Rarefaction resulted in a final total of 581,950 reads (1,138 unique taxa with 226 samples). This preprocessed, contaminant removed, and rarefied data were then subject to statistical analysis and visualization with a variety of packages including phyloseq (45), vegan (46), and ggplot2 (47). An α = 0.05 was used for all analyses. A feature table containing ASV counts, corresponding DNA sequence file, and the taxonomy file are available as supplementary files (Supplementary Files 1A, B, and 2). A metadata file containing treatment types and corresponding diversity data is also available (Supplementary File 1C).

We first examined probiotic persistence. This was done using post-rarefaction ASV counts to visualize the amount and presence of the probiotic ASVs in each treatment and control group over time. To identify the ASVs that corresponded to the probiotic used in exposure, we used Geneious 10.2.2 with a custom Blast to return the ASVs that matched at 100% sequence similarity to their respective 16S rRNA gene sequence (28). Only one ASV matched at 100% sequence similarity for each probiotic and is hereafter considered the same taxon as the probiotic applied bacteria. Significance of changes in probiotic persistence over time was examined using linear mixed-effects models, with the probiotic ASV sequence count as a response variable, day as a linear fixed effect, and frog ID as a random effect (Jupyter Notebook, Analyses/Statistics). In the case of the model used for the probiotic cocktail, the parameters approached the boundary of the parameter space (i.e., singularity) but functioned as expected, indicating overall stability.

Next, we examined microbial community alpha diversity over time and between treatments. All alpha diversity examinations were completed using ASV richness. ASV richness was also examined for a community subset of known bacterial taxa with anti-*Bd* inhibitory properties (48; an updated version of the Woodhams database with only strongly inhibitory bacteria was used, Strict_June15.2020 update, personal communication from reference [31]) with a correction made to examine the community minus the probiotics for a corresponding ASV relative abundance analysis (total anti-Bd ASV read counts divided by total reads) and richness analysis. For both analyses, we first determined if there were any baseline differences in ASV richness at day 0, prior to probiotic exposure. This was done using analysis of variance (ANOVA) and Tukey’s post hoc tests, where applicable. To compare alpha diversity changes over time after the probiotic treatment, we used linear mixed effects models with bacterial ASV richness as the response variable, treatment and day as linear fixed effects, and frog ID as a random effect. These models allowed us to determine if probiotic exposure changed the bacterial community structure among treatments at week 1 (*n* = 16–18 individuals/treatment; Table 2), week 2 (*n* = 6 resampled individuals/treatment; Table 2), and week 3 (*n* = 6 resampled individuals/treatment; Table 2). Richness was log_10_ transformed to meet assumptions of normality. To determine the significance of the day and treatment fixed effects, Wald’s test was used. This was followed by an examination of pairwise differences between treatment groups using estimated marginal means.

Following alpha diversity analysis, beta diversity was examined. Rarefied ASVs were also used in beta-diversity calculations. Between treatment community differences were examined at each time point using both the Bray-Curtis and Jaccard dissimilarity metrics for beta-diversity using permutational analysis of variance (PERMANOVA). Differences for both metrics were visualized using principal coordinate analysis (PCoA). In addition to the PERMANOVAs and PCoAs, beta dispersions (Jaccard and Bray-Curtis) between treatment groups by time point were also examined using the betadisper function in vegan.

### Data analysis and statistics: targeted RT-qPCR

Immunity gene expression analysis utilized the 2^−ΔΔCT^ method for normalization and statistical comparison (42). A correlation analysis on this data were first completed to better understand sample independence (Table S8). This was followed by visual inspection of the normalized expression data, which indicated data that violated assumptions of normality (Jupyter Notebook, Analyses/Statistics). This was ameliorated by using non-parametric Kruskal-Wallis tests and Dunn’s post hoc tests to compare non-correlated genes and genes of interest at both week 1 and week 3. Time groups were analyzed separately as the different time groups were composed of different individuals (i.e., no repeat sampling due to animal euthanasia for tissue collection).

Lastly, probiotic ASV sequence count per respective treatment group was examined as a function of immune expression data for *FOXP3,* colony stimulating factor-1 (*CSF1),* and interleukin-10 (*IL10*) using linear models. Each individual probiotic ASV sequence count was the response variable, and the explanatory variables were the three non-correlated genes of interest (*CSF1, FOXP3,* and *IL10*). Models were analyzed for each probiotic treatment separately. For the cocktail, each unique probiotic ASV was isolated and compared in separate analyses (e.g., *Pseudomonas* RSB5.4 [P1] in cocktail [P4] was subset and analyzed separately from *Stenotrophomonas* THA2.2 [P2] in cocktail [P4]). Both log_10_ transformation and no transformation were utilized. Other genes of interest were excluded from this analysis due to issues with collinearity. QQ plots were examined to verify model fit.

### Data analysis and statistics: transcriptomics

Read mapping and transcript quantification were conducted using the nf-core/rnaseq pipeline (v. 3.14.0-gb89fac3) in the Nextflow (v. 24.04.4) workflow manager (49, 50). The STAR-Salmon sub-workflow was used (51, 52), the *X. laevis* genome assembly (v. 10.1) and annotations (v. 10.17) were used as a mapping reference (53) and default values were used for all other settings. Protein sequences were extracted from the genome with gffread (v.0.12.7; 54) and annotated with eggNOG-mapper (v.2.1.5; 55) to derive gene names and gene ontology (GO) terms.

Differentially expressed genes (DEGs) were identified using the DESeq2 (v. 1.34.0) R package (56). Samples from each timepoint (weeks 1 and 3) were separated prior to DESeq2 analysis because intra-group variation was markedly different between timepoints (as recommended by the DESeq2 vignette). Genes with fewer than 10 reads for at least 12 or 6 samples for weeks 1 and 3, respectively (representing the respective group sizes), were removed from further analysis. DEGs were identified between each treatment and the control for each time point (with corrected *P*-values < 0.05 considered significant). Overlap between each set of DEGs was visualized using UpSet plots with the UpSetR (v.1.4.0) R package (57). Significantly enriched biological process GO terms were identified for each set of DEGs (including both significantly up and down-regulated genes) using the topGO (v. 2 46.0) R package (58).

Associations between gene expression and bacterial ASV sequence count were investigated using a weighted gene co-expression network analysis (WGCNA) approach (59). Since large numbers of samples and variation in associations are required for network inference, samples across all treatments, including controls, were used in a single analysis. The final network represents the variation of gene expression-bacterial ASV sequence count associations that is possible for *X. laevis* skin. Gene-level expression data were first transformed using the variance-stabilizing transformation in DESeq2, and only genes with 10 or more reads for at least 6 samples were retained. Genes were then clustered into co-expression modules using the WGCNA (60) package (v. 1.72-5) in R using a soft power threshold of 3, a maximum block size of 15,000, and a signed topological overlap matrix. Modules were functionally annotated by calculating the proportion of their genes containing each of the level one biological process GO terms. Bacterial ASV sequence count was agglomerated to the genus level, filtered to retain only genera present in 50% of samples, and converted to relative abundance using the R package phyloseq (v.1.41.1; 45). Gene module expression and bacterial relative abundance were then combined, and Spearman’s correlation tests were performed between all gene modules and bacteria, with only correlations with a (FDR-corrected) *P*-value < 0.05 being retained. These were input into Gephi (v.0.10.1; 61) for network visualization.

## RESULTS

### Probiotic persistence

**Fig 1:**
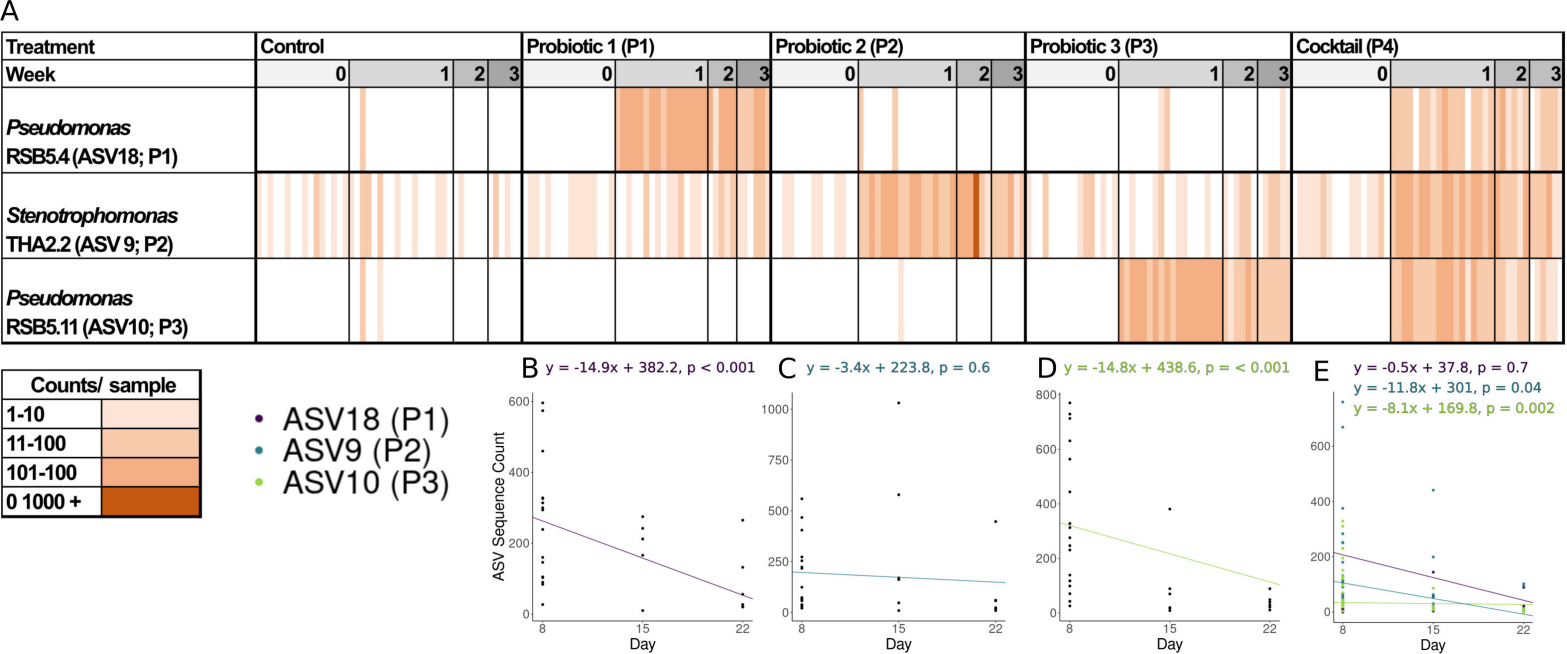
Probiotic persistence. (A) Heatmap abundances of ASV sequence counts per sample by week 0 (day 0; prior to probiotic inoculation), week 1 (day 8), week 2 (day 15), and week 3 (day 22). Sample range by color shade is given in the corresponding legend. Scatterplot of probiotic sequence counts for each probiotic treatment and corresponding linear mixed-effects model equation for (B) ASV18 (Pseudomonas RSB5.4 [P1]), (C) ASV9 (Stenotrophomonas THA2.2 [P2]), (D) ASV10 (Pseudomonas tolaasii RSB5.11, P3), and (E) the three-probiotic cocktail.

In all probiotic exposure treatments, the probiotics generally persisted over the 22-day experiment (Fig. 1). For frogs exposed to *Pseudomonas* RSB5.4 (P1), *Stenotrophomonas* THA2.2 (P2), and *Pseudomonas tolaasii* RSB5.11 (P3), all treatment groups maintained detectable amounts of the probiotic until the end of the experiment. *Stenotrophomonas* THA2.2 (P2) was detected as a common symbiont on *X. laevis* in all treatment groups including controls, and with probiotic exposure, *Stenotrophomonas* THA2.2 (P2). ASV sequence count increased in the P2 (monoculture exposure) and P4 (cocktail exposure) treatments.

Different outcomes of probiotic persistence occurred over 22 days depending on the strain and monoculture versus cocktail application. *Pseudomonas* RSB5.4 (P1; Fig. 1B) and *Pseudomonas tolaasii* RSB5.11 (P3; Fig. 1D) significantly declined over time in sequence count (LMM: *P* < 0.001), whereas *Stenotrophomonas* THA2.2 (P2; Fig. 1C) remained stable over time (LMM: *P* = 0.6). However, in the three-probiotic cocktail (P4; Fig. 1E), *Pseudomonas* RSB5.4 (P1; Fig. 1E; LMM: *P* = 0.7) remained stable over time, whereas *Stenotrophomonas* THA2.2 (P2; Fig. 1E; *P* = 0.04) and *Pseudomonas tolaasii* RSB5.11 (P3; Fig. 1E; *P* = 0.002) significantly declined in sequence count.

### Probiotic inoculation effect on bacterial ASV richness and relative abundance

Prior to any probiotic exposure, day 0 ASV richness was similar among treatments (ANOVA: F_4,75_ = 1.56; *P* = 0.194). Following probiotic exposure, ASV richness remained similar among treatments (Wald: W = 4.26; df = 4, *P* = 0.37) but changed over time, with a slight but significant increase with time (Wald: W = 21.07; df = 1, *P* < 0.001) (Fig. S2).

**Fig 2:**
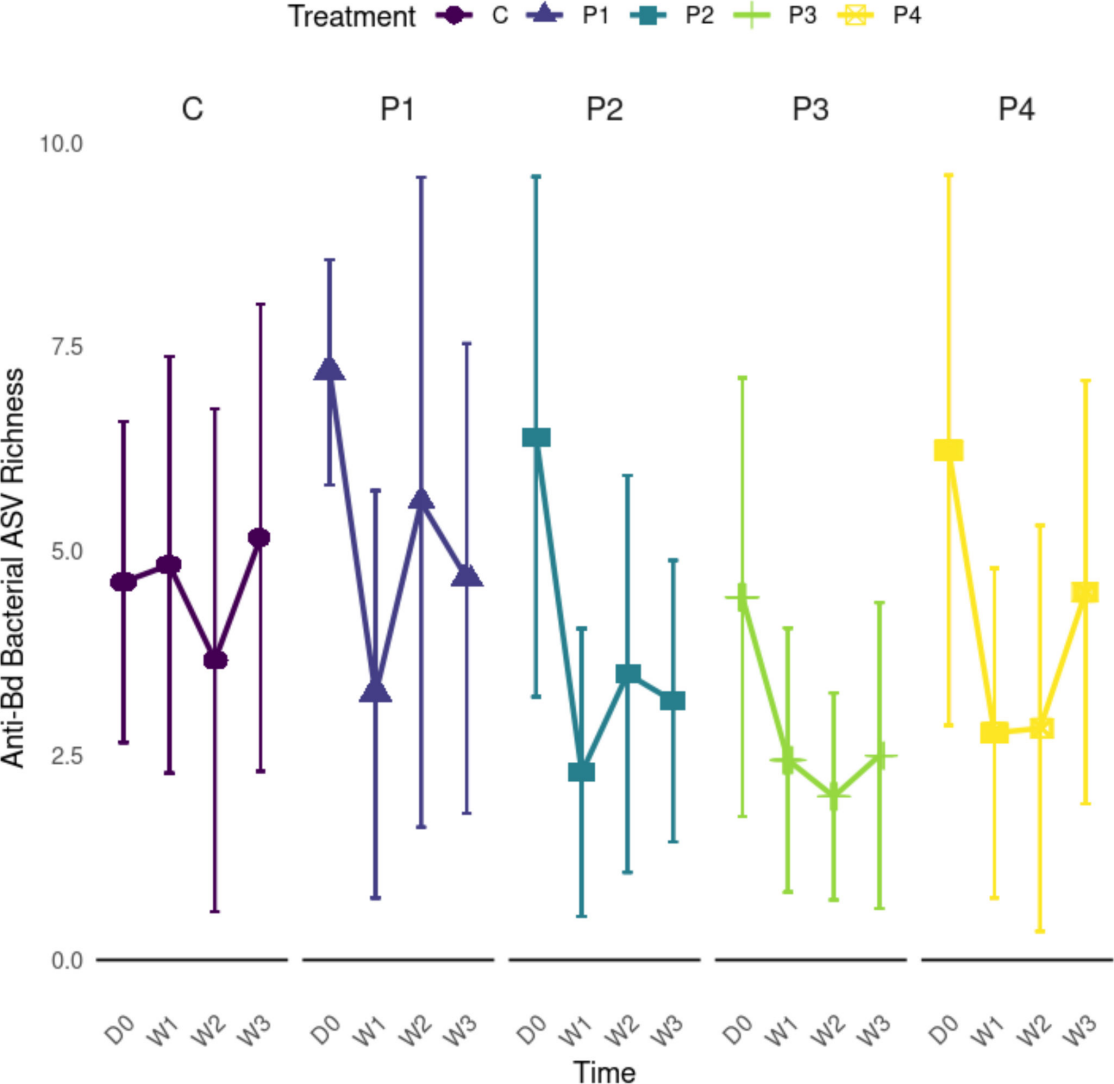
Line plots of anti-*Bd* bacterial ASV richness, by treatment and timepoint. All treatment groups at the beginning of the experiment pre-inoculation, were similar at day 0 (D0). All treatments at week 1 post-inoculation (W1), week 2 post-inoculation (W2), and week 3 post-inoculation (W3) also had similar or slightly decreasing anti-*Bd* bacterial ASV richness over time, with P4 having slightly higher richness (data shown is excluding observed probiotics by respective individuals at each timepoint). Treatments are matched by color and are coded as follows: C = no-probiotic control, P1 = *Pseudomonas* RSB5.4, P2 = *Stenotrophomonas* THA2.2, P3 = *Pseudomonas* RSB5.11, and P4 = cocktail. Error bars at each point represent plus or minus one standard deviation.

Probiotic effects on the ASV richness of known anti-*Bd* bacteria were also examined (Fig. 2). Prior to probiotic exposure, a significant difference was observed between treatments (ANOVA: F_4,75_ = 2.761; *P* = 0.0337). Specifically, *Pseudomonas* RSB5.4 (P1) and *Pseudomonas tolaasii* RSB5.11 (P3) treatments differed initially, with treatment *Pseudomonas* RSB5.4 (P1) having a significantly higher anti-*Bd* bacterial ASV richness pre-probiotic exposure than treatment *Pseudomonas* RSB5.11 (P3) (*P* = 0.0241) by chance. Following probiotic exposure, anti-*Bd* bacterial richness (inclusive of the probiotics) had a significant treatment effect (Wald: W = 18.56; *P* < 0.001), but no significant time effect (Wald: W = 2.25; *P* = 0.13). The probiotic cocktail (P4) had significantly higher anti-*Bd* bacteria richness (inclusive of probiotics) compared to treatments *Stenotrophomonas* THA2.2 (P2) (marginal means pairwise comparison *Stenotrophomonas* THA2.2 (P2)-cocktail (P4): *P* = 0.0037) and *Pseudomonas* RSB5.11 (P3) (marginal means pairwise comparison *Pseudomonas tolaasii* RSB5.11 (P3)-cocktail (P4): *P* = 0.0093) (Table S1). Anti-*Bd* bacteria richness was then corrected for the added probiotics (subtracting 0–3 for each individual frog and time point depending on presence/absence of *Pseudomonas* RSB5.4 [P1], *Stenotrophomonas* THA2.2 [P2], and *Pseudomonas tolaasii* RSB5.11 [P3]) and still showed a significant treatment effect post-exposure (Wald: W = 16.558; *P* = 0.002) but did not show a significant overall time effect (Wald: W = 2.933; *P* = 0.0868). When compared to the control group, the control (C)-*Stenotrophomonas* THA2.2 (P2) and control (C)-*Pseudomonas tolaasii* RSB5.11 (P3) marginal means pairwise treatment comparisons indicated that probiotic treatment caused anti-*Bd* bacteria richness to be significantly reduced (*P* < 0.05) (Table S2).

**Fig 3:**
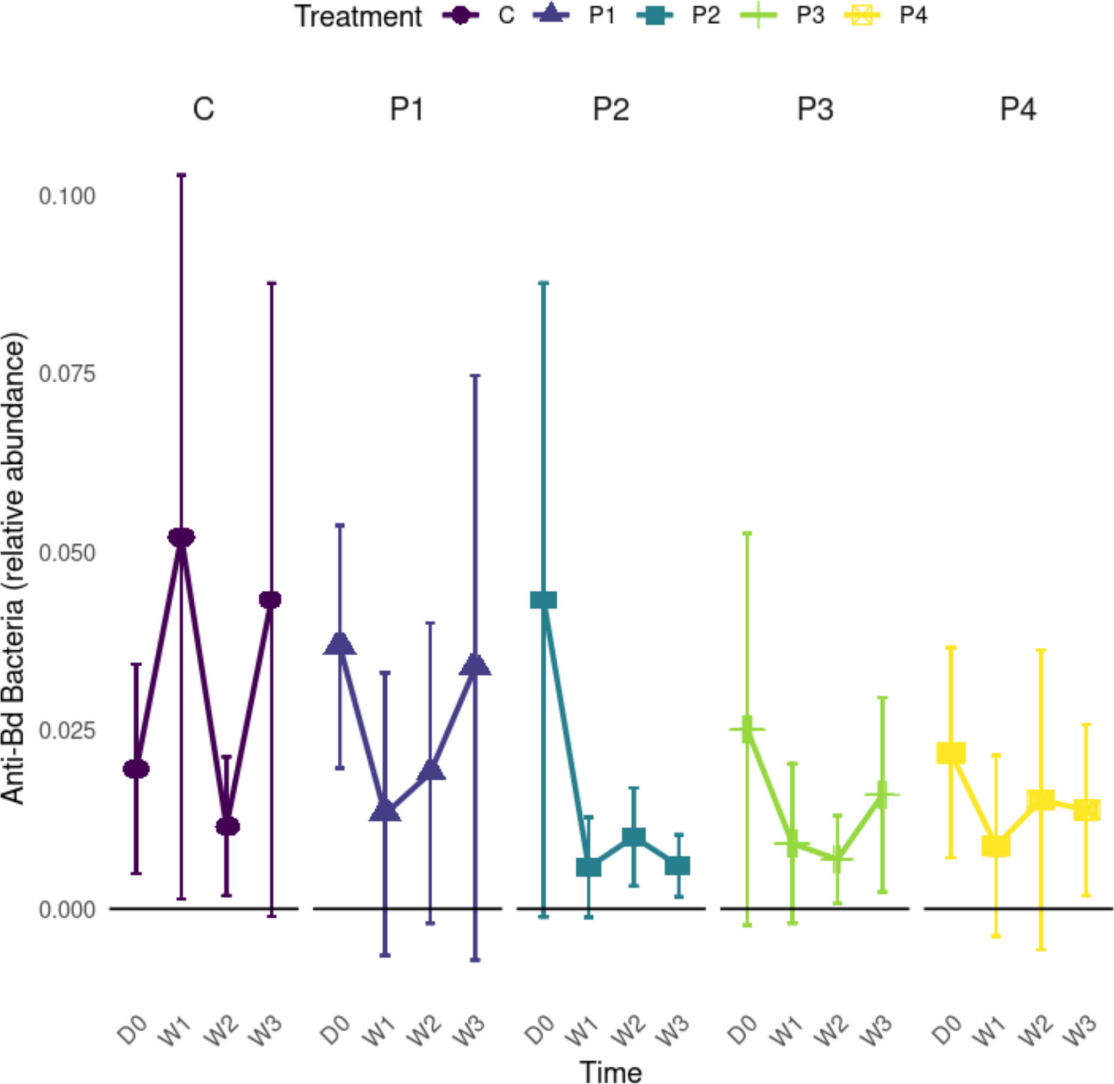
Line plots of corrected ASV relative abundance of anti-*Bd* bacteria, by treatment and timepoint. All treatment groups exhibited a lower relative abundance of anti-*Bd* bacteria over time as compared to the control (data shown is excluding observed probiotics by respective individuals at each timepoint). Treatments are matched by color and are coded as follows: C = no-probiotic control, P1 = *Pseudomonas* RSB5.4, P2 = *Stenotrophomonas* THA2.2, P3 = *Pseudomonas tolaasii* RSB5.11, and P4 = cocktail. Time points are given for each treatment and are coded as follows: D0 = day 0, W1 = week 1, W2 = week 2, and W3 = week 3. Error bars at each point represent plus or minus one standard deviation.

We then tested for any treatment and time effects on the relative abundance of anti-*Bd* bacteria ASVs (after removing probiotic applied sequences from analyses; Fig. 3). Prior to probiotic exposure, anti-*Bd* bacterial ASV relative abundance was similar among treatments (ANOVA: F_4,75_ = 2.452; *P* = 0.0532). Following probiotic exposure, anti-*Bd* bacterial ASV relative abundance differed among treatments (Wald: W = 38.79; df = 4, *P* < 0.001), but not time (Wald: W = 0.278; df = 1, *P* = 0.598). Probiotic treatment caused a significant reduction in anti-*Bd* bacteria ASV relative abundance for all treatment groups when compared to the control group (C) with no probiotics (Table S3).

### Probiotic inoculation effect on microbial community beta-diversity

**Fig 4:**
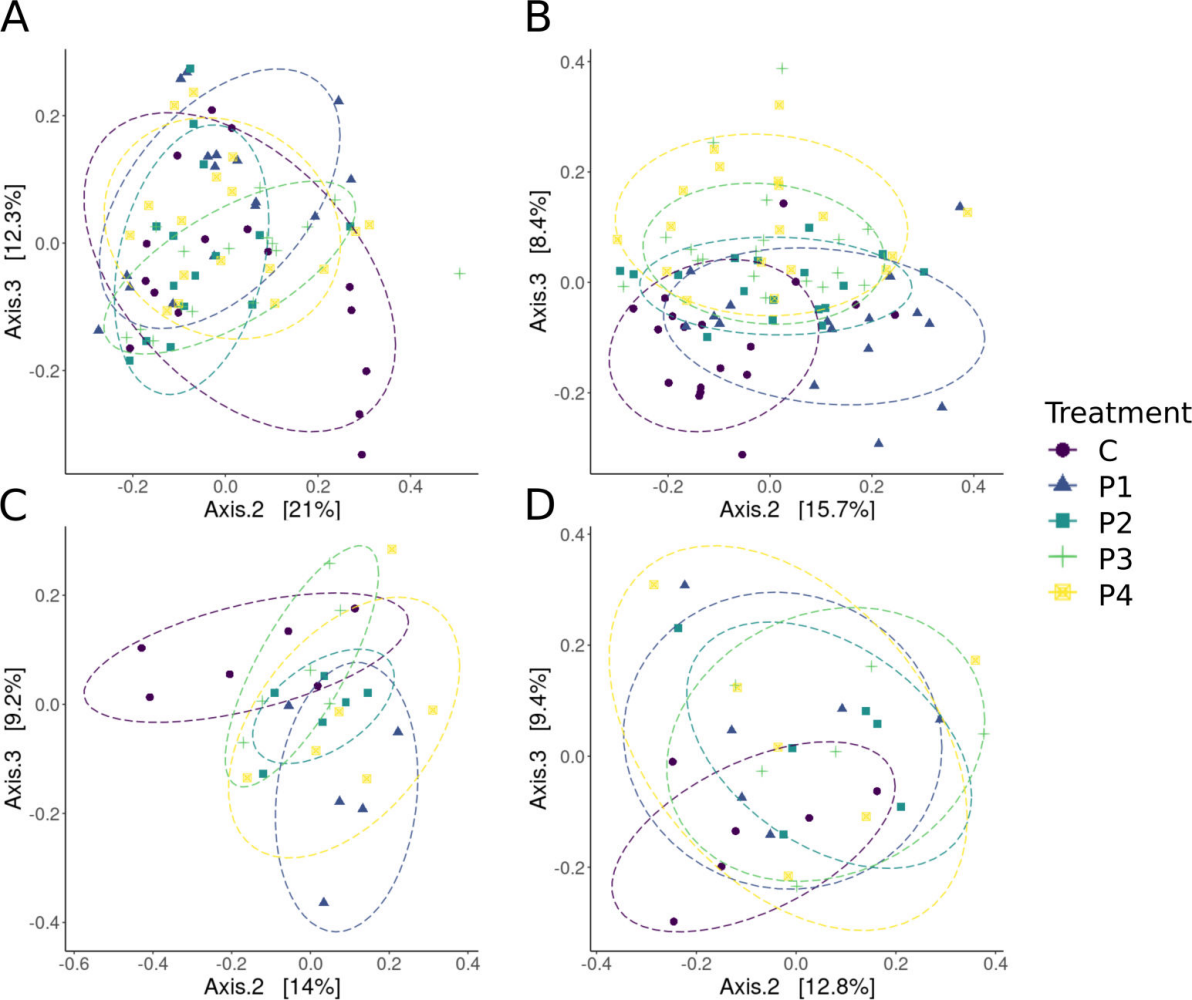
Principal coordinate analyses (axes 2 and 3; shown to visualize treatment effects) of microbial community beta diversity using the Bray-Curtis dissimilarity metric, by time point. Data ellipses show an 80% confidence and assume a multivariate t-distribution. The treatment group is given by color and shape in the legend. (A) All treatment groups at the beginning of the experiment, pre-inoculation. (B) All treatments at week 1 post-inoculation. (C) All treatments at week 2 post-inoculation. (D) All treatments at week 3 post-inoculation. Treatments are matched by color across panels and are coded as follows: C = no-probiotic control, P1 = *Pseudomonas* RSB5.4, P2 = *Stenotrophomonas* THA2.2, P3 = *Pseudomonas tolaasii* RSB5.11, and P4 = cocktail.

The bacterial community beta-diversity differed among time points (Fig. S3 and S4) and among treatments within a given time point (Fig. 4). We observed large compositional shifts in all treatments at week 1 compared to day 0, followed by continual but more gradual shifts in the bacterial community composition at weeks 2 and 3. All treatments, including controls, experienced these shifts, suggesting a continually changing state of the *X. laevis* skin microbiome. Prior to probiotic exposure, differences in skin bacterial community composition among treatments using the Bray-Curtis (PERMANOVA: F_4,75_ = 2.56; *P* ≤ 0.05; Table S4) and Jaccard dissimilarity metrics (PERMANOVA: F_4,75_ = 1.49; *P* ≤ 0.05; Table S6) were observed by chance. However, in post hoc comparisons, no treatments were found to differ with the Bray-Curtis metric (post hoc: F(model) = 1.24–3.52; df = 1; *P* > 0.05; Table S5). With the Jaccard metric, *Stenotrophomonas* THA2.2 (P2) and *Pseudomonas* RSB5.4 (P1) treatments differed from control (C) and cocktail (P4), and *Pseudomonas* RSB5.4 (P1) also differed from *Pseudomonas tolaasii* RSB5.11 (P3) (*P* ≤ 0.05; Table S7), indicating that unintentional treatment effects were present but subtle prior to exposure. At week 1 following probiotic exposure, most treatments differed from one another using the Bray-Curtis metric (PERMANOVA: F_4,82_ = 4.50; *P* ≤ 0.05; post hoc: *P* ≤ 0.05; Tables S4 and S5) except *Stenotrophomonas* THA2.2 (P2) and *Pseudomonas tolaasii* RSB5.11 (P3) did not differ from cocktail (P4), and all treatments differed in pairwise comparisons in Jaccard (PERMANOVA: F_4,82_ = 2.02; *P* < 0.05; post hoc: *P* ≤ 0.05; Tables S6 and S7). By week 2, treatment effects were still detected for Bray-Curtis (PERMANOVA: F_4,24_ = 1.62; *P* = 0.009; Table S4) and Jaccard metrics (PERMANOVA: F_4,24_ = 1.37; *P* ≤ 0.05; Table S6). In post hoc comparisons, no pairwise treatments were found to differ with the Bray-Curtis metric (*P* > 0.05; Table S5), and with the Jaccard metric, cocktail (P4) differed from control (C), and *Stenotrophomonas* THA2.2 (P2) differed from *Pseudomonas tolaasii* RSB5.11 (P3) (*P* ≤ 0.05; Table S7). Finally, in week 3, significant treatment effects were no longer present for the Bray-Curtis metric (PERMANOVA: F_4,25_ = 1.17; *P* = 0.154; Table S4) but were observed for the Jaccard metric (PERMANOVA: F_4,25_ = 1.29; *P* ≤ 0.05; Table S6). In post hoc comparisons for the Jaccard metric, significant differences between treatments were observed between control (C) and both *Stenotrophomonas* THA2.2 (P2) and cocktail (P4). Significant differences were also seen between *Pseudomonas* RSB5.4 (P1) and *Stenotrophomonas* THA2.2 (P2) (*P* ≤ 0.05; Table S7).

Beta dispersion for the Jaccard metric was also examined between treatment groups by timepoint. Significant differences in beta dispersion were not seen in week 1, week 2, and week 3 (F_4,24–82_ = 1.28–2.10; *P* > 0.05). Significant differences were observed in beta dispersion prior to probiotic exposure (D0; F_4,75_ = 5.49; *P* < 0.05). Follow-up pairwise testing indicates that these significant differences (*P* < 0.05) were present between *Pseudomonas* RSB5.4 (P1) and the following: control (C), *Stenotrophomonas* THA2.2 (P2), and *Pseudomonas tolaasii* RSB5.11 (P3). For beta dispersion analyses using the Bray-Curtis metric, no significant differences were observed between treatments for all four timepoints (F_4,24–82_ = 0.71–1.78; *P* > 0.05).

### Targeted RT-qPCR expression analysis

**Fig 5:**
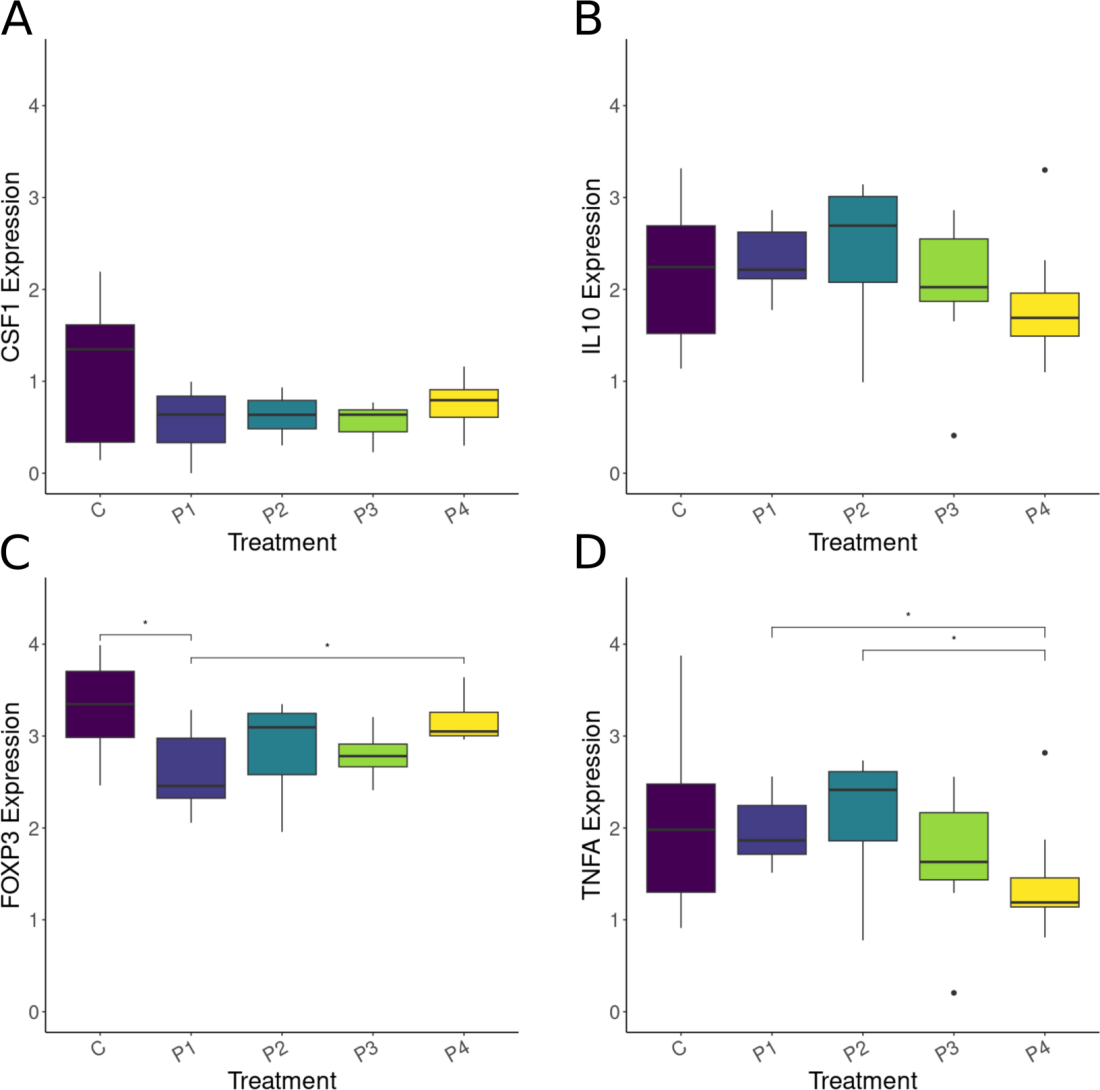
Boxplots of 2^−ΔΔCT^ *GAPDH* normalized expression data at week 1 for (A) *CSF1*, (B) *IL10*, (C) *FOXP3*, and (D) *TNFA*. The expression (y-axis) scale has been log_10_ normalized for purposes of visualization only. Bars with * indicate a significant difference of *P* < 0.05. Treatments are matched by color across panels and are coded as follows: C = no-probiotic control, P1 = *Pseudomonas* RSB5.4, P2 = *Stenotrophomonas* THA2.2, P3 = *Pseudomonas tolaasii* RSB5.11, and P4 = cocktail.

Gene expression analysis of eight hallmark immune-related genes was conducted at key times post-probiotic treatment (Fig. 5). We first tested the correlational structure of the eight examined immune genes, with low correlation genes having independent and analyzable expression patterns. We found high correlations (Pearson Correlation >0.9; Table S8) for interleukin-10 (*IL10*; immune suppression [62]), interleukin-34 (*IL34;* macrophage growth factor [63])*,* transforming growth factor-beta (*TGFB;* immune suppression and fibrosis [64])*,* toll-like receptors 2 and 6 (*TLR;* pathogen recognition [65])*,* and tumor necrosis factor-alpha (*TNFA;* proinflammatory [66]). The low correlation genes were identified as colony stimulating factor-1 (*CSF1;* macrophage growth factor [63]) and forkhead box P3 (*FOXP3:* marker of T regulatory cells [67]). Two genes of biological interest were also included in our analysis, *IL10* and *TNFA*. These were included in the individual analyses because *TNFA* is a pro-inflammatory cytokine while *IL10* is typically involved in immune suppression, thus the two are not expected to exhibit co-expression or overlapping biological function, even though they exhibited correlated expression patterns in our analysis.

Probiotic treatment effects were examined on *CSF1*, *IL10*, *FOXP3*, and *TNFA* gene expression. At week 1, no significant differences were observed among treatments for *CSF1* expression (*H* = 5.72; df = 4; *P* = 0.22) and a near significant effect of *IL10* expression was observed (Kruskal-Wallis: *H* = 9.28; *P* = 0.054; df = 4). Conversely, *FOXP3* expression (*H* = 11.98; df = 4; *P* = 0.018) and *TNFA* expression differed among treatments (*H* = 12.82; df = 4; *P* = 0.012). Specifically, the exposure of frogs to *Pseudomonas* RSB5.4 (P1) resulted in lowered *FOXP3* expression compared to control (C) frogs (*P* = 0.0288; Table S9) and to frogs treated with the cocktail (P4; post hoc: *P* = 0.0463; Table S9). Distinct probiotic treatments exhibited disparate effects on *TNFA* expression. Specifically, the cocktail (P4) caused a significant reduction in *TNFA* expression compared to expression levels seen in *Pseudomonas* RSB5.4 (P1) and *Stenotrophomonas* THA2.2 (P2) (post hoc: *P* ≤ 0.05; Table S10). At week 3, no significant differences were observed among treatment groups for *CSF1*, *IL10*, *FOXP3*, or *TNFA* expression (Kruskal-Wallis rank sum test: *H* = 2.71, *P* = 0.61; *H* = 3.93, *P* = 0.42; *H* = 4.99, *P* = 0.29; *H* = 4.57, *P* = 0.33; respectively; df = 4 for all tests).

Finally, gene expression was examined in relation to the counts of each probiotic ASV in their respective treatments. We found no linear relationship between probiotic ASV sequence counts and *FOXP3*, *IL10*, and *CSF1* gene expression for both the log_10_ transformed and untransformed data (*P* > 0.05; Jupyter Notebook, Analyses/Statistics).

### Transcriptomic analysis

**Fig 6:**
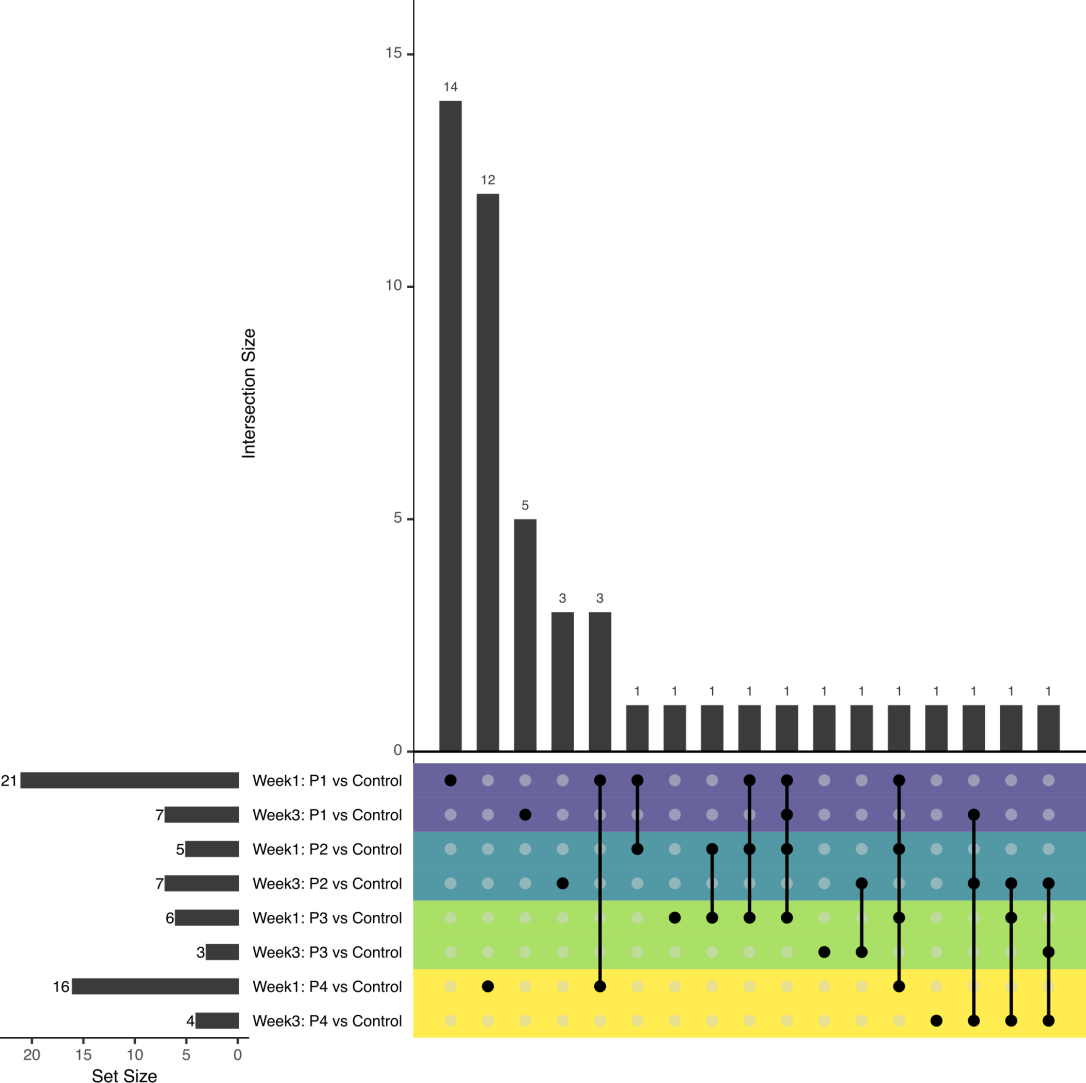
Upset plot showing overlap between each set of differentially expressed immune-related genes. Horizontal bars show the size of each set, and vertical bars show the size of each intersection between sets (denoted by black points within the central grid). The rows of the central grid are colored by test treatment. Treatments are coded as follows: C = no-probiotic control, P1 = Pseudomonas RSB5.4, P2 = Stenotrophomonas THA2.2, P3 = Pseudomonas tolaasii RSB5.11, and P4 = cocktail.

To expand on our immune gene expression analysis, we performed RNA-seq of control and probiotic-exposed frog skins. This analysis produced between 38 and 75 million read pairs per sample, which were mapped to the *X. laevis* genome at a rate of 81.5% to 86.3% (Supplementary File 1D). We tested 22,080 genes (post-filtering) for differential expression across all treatments with 288 identified as DEGs in at least one treatment compared to controls (Supplementary File 1E). The majority of DEGs were probiotic treatment-specific for all DEGs (Fig. S5) and for immune-related DEGs specifically (Fig. 6). We then determined if DEGs were enriched for specific GO terms, including immune-related GO terms (Fig. S6; Supplementary File 1F).

At both week 1 and week 3, probiotic application caused changes to the expression of relatively few subsets of immune-related genes, with limited overlap between probiotic sets (Fig. 6; Fig. S7; Supplementary File 1G). The treatments *Pseudomonas* RSB5.4 (P1) and cocktail (P4), which both contained *Pseudomonas* RSB5.4, elicited higher immune-related gene expression changes at week 1 (*Pseudomonas* RSB5.4 [P1] = 21 DEGs; cocktail [P4] = 16) compared to the other treatments (*Stenotrophomonas* THA2.2 [P2] = 5; *Pseudomonas tolaasii* RSB5.11 [P3] = 6). After 1 week of treatment, both *Pseudomonas* RSB5.4 (P1) and cocktail (P4) treatments resulted in increased expression of the antiviral gene encoding interferon-induced transmembrane protein 3 (*IFITM3*). After 1 week of treatment, *Pseudomonas* RSB5.4 (P1) but not cocktail (P4) also resulted in increased expression of the antiviral gene encoding an interferon-induced protein with tetratricopeptide repeats 5 (*IFIT5*). Additionally, *Pseudomonas* RSB5.4 (P1) treatment resulted in elevated expression of a handful of genes associated with innate (*C3, SEPINE1*) and adaptive (*LCK, CD7, PRF1*) arms of the immune response. Cocktail (P4) was also the only treatment to exhibit a significant reduction in immunoglobulin J (*IGJ*) expression compared to control (C) at week 1. Also, *Pseudomonas tolaasii* RSB5.11 (P3) was the only treatment to display increased interleukin-17 (*IL17C*) expression at week 1. Interestingly, animals treated for 3 weeks with *Pseudomonas* RSB5.4 (P1), *Stenotrophomonas* THA2.2 (P2), or the cocktail (P4) probiotics all possessed elevated expression of the ficolin-2 (*FCN2*) gene, which is associated with the lectin pathway of complement activation (68). Notably, 3 weeks of treatment with *Stenotrophomonas* THA2.2 (P2) and *Pseudomonas tolaasii* RSB5.11 (P3) resulted in elevated expression of the gene encoding complement factor B, while *Pseudomonas tolaasii* RSB5.11 (P3) resulted in decreased interleukin-12 (*IL12B*) expression, and cocktail (P4) resulted in increased C2 complement gene expression.

For GO terms, only a few were consistently enriched within each probiotic treatment over time (Fig. S6; Supplementary File 1F). It is unclear to what extent, if any, these terms are involved in *X. laevis* immune responses. However, we did observe signatures of probiotic application in a small number of GO terms. At week 1, the GO terms “negative regulation of monocyte differentiation,” “negative regulation of myeloid cell differentiation,” and “definitive hemopoiesis” were enriched for all four treatment-control comparisons at week 1 and the term “opsonization” was enriched at week 3 (Supplementary File 1F). The only significant GO term found in probiotic treatments containing *Pseudomonas* (*Pseudomonas* RSB5.4 [P1], *Pseudomonas tolaasii* RSB5.11 [P3], and cocktail [P4]) and not in the *Stenotrophomonas* THA2.2 (P2) treatment was the GO term “negative regulation of hemopoiesis” at week 1.

**Fig 7:**
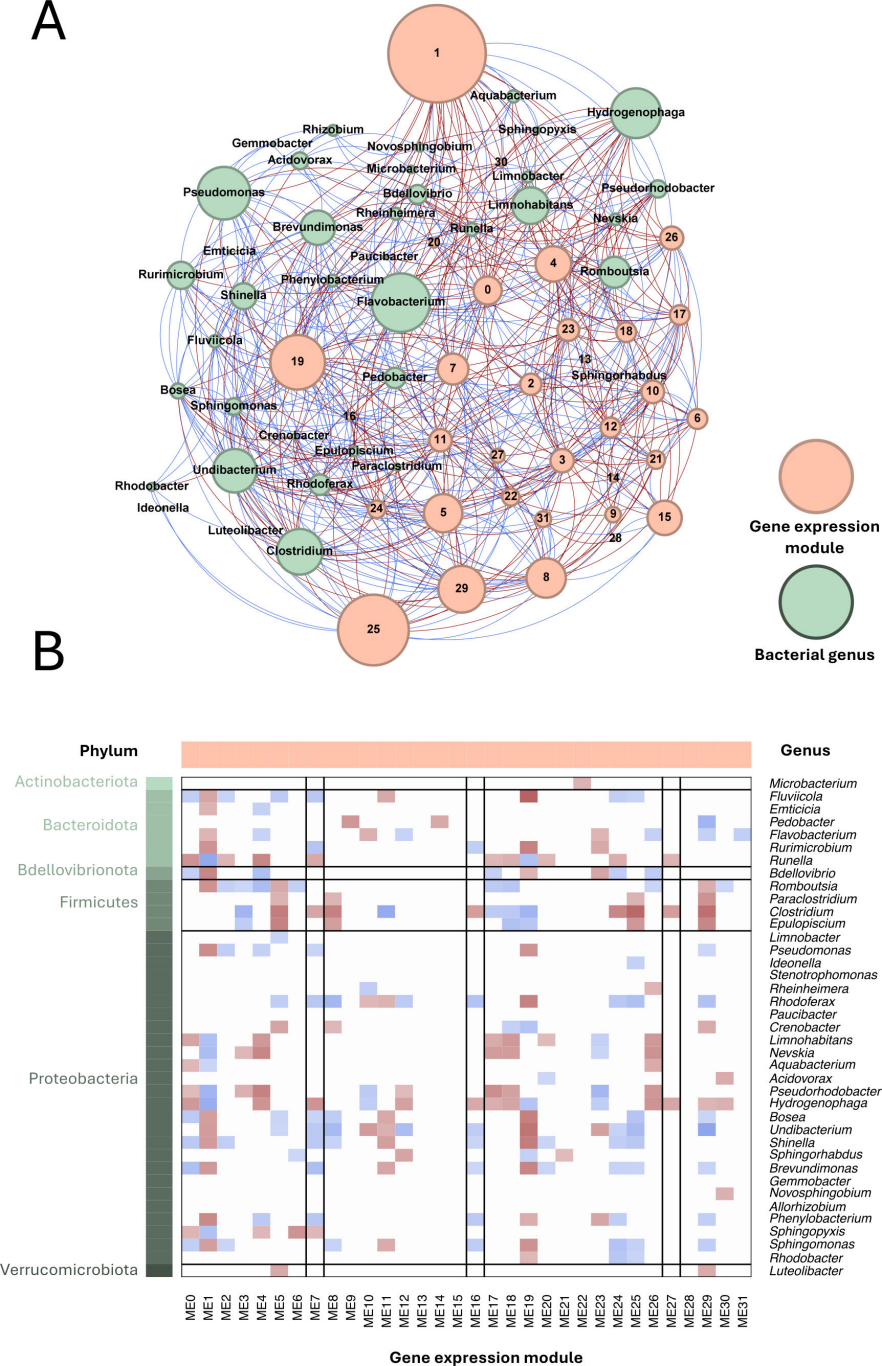
An association network of proportional abundance of bacterial genera and gene expression modules across all treatments (A). Nodes are colored by data type (bacterial genus/expression module) and their size is scaled by betweenness centrality. Edge color indicates direction of association (red = negative, blue = positive). The heatmap shows correlations (red = negative, blue = positive) between gene expression modules (B) and bacterial genera. Matrix color shows direction (as in panel A) and strength of correlation; non-significant correlations are set to zero (white). Bacteria are ordered by phylum. Vertical lines highlight the modules with the highest proportion of genes annotated with the “immune system process” GO term.

We assessed overall associations between gene expression and bacterial sequence counts in *X. laevis* using network analysis (WGCNA; all treatments combined to build robust networks). The genes were partitioned into 32 modules in the WGCNA analysis. The majority of modules were significantly associated with sequence counts representing at least one bacterial genus (Fig. 7). Three modules contained a particularly high proportion of immune-related genes (Fig. S5) and two of these (ME7 and ME16) had both positive and negative associations with multiple bacterial taxa. Notably, ME7 and ME16 had generally positive associations with Proteobacteria taxa and ME7 with *Pseudomonas* specifically. Interestingly, while *Pseudomonas* (introduced in treatments *Pseudomonas* RSB5.4 [P1], *Pseudomonas tolaasii* RSB5.11 [P3], and cocktail [P4]) was significantly associated with multiple gene expression modules, *Stenotrophomonas* (introduced in treatments *Stenotrophomonas* THA2.2 [P2] and cocktail [P4]) was not significantly associated with any gene modules (Fig. 7B). The lack of host-bacterial correlation for *Stenotrophomonas* may be reflected in the low number of differentially expressed genes for the *Stenotrophomonas* THA2.2 (P2) contrast (Fig. 6) and may relate to this probiotic being a common commensal on *X. laevis* used in the experiment (Fig. 1).

## DISCUSSION

Here, we hypothesized that probiotic exposure with three previously identified *Bd* inhibitory bacteria would result in skin colonization and post-exposure persistence on the model frog species, *X. laevis*. We demonstrated that probiotic exposure results in colonization and persistence of applied probiotics for at least three weeks, with probiotic strains showing different persistence patterns in monoculture versus mixed cocktail application. We also hypothesized that probiotic exposure in *X. laevis* would alter both the resident bacterial community and expression profiles of genes involved in the *X. laevis* innate and adaptive immune response. We showed alterations of both the bacterial community and immune gene expression patterns. Bacterial community changes at week 1 and differences in immune gene expression at week 1 and week 3 were signatures of all probiotic applications.

Probiotic persistence was observed for all probiotic treatments over the course of the experiment, albeit with a decline in ASV sequence count for *Pseudomonas* RSB5.4 (P1) and *Pseudomonas tolaasii* RSB5.11 (P3) when applied independently. In the probiotic cocktail treatment, a decline in ASV sequence count was seen for *Stenotrophomonas* THA2.2 (P2) and *Pseudomonas tolaasii* RSB5.11 (P3), indicating differential persistence effects of probiotics when introduced independently as opposed to a mixture. This is an important *in vivo* finding in *X. laevis* and corroborates findings *in vitro* (69) and *in vivo* in *A. zeteki* (25) of anti-*Bd* cultures used in multi-species probiotic treatments enhancing or reducing the effects of other members. The differential effects seen in the probiotic cocktail treatment also have important similarities to results of probiotic studies in *Rana sierrae,* where an experimental anti-*Bd* multi-species bacteria consortium mediated host peptide reduction (16). The effects of single versus multi-species probiotics should therefore be considered in future treatment designs. Whether one probiotic is deterministically selected over the other through a host sorting mechanism via host factors such as immune selection (70) and open ecological niches in the microbial community (71) or is favored through random assembly effects (72) remains unclear. Priority effects encompassing the timing of microbial community assembly in tadpoles have also recently been shown to influence probiotic prevalence (73). Such timing may have important causal interactions with amphibian immune responses and should therefore be explored in future work. Additionally, the mathematical prediction of probiotic sorting may follow theoretical approaches. Specifically, results presented here might be explained by a complex interaction of ecological and evolutionary processes, to include host-orchestrated species sorting via the host-immune system and microbiome (74).

We found that the two *Pseudomonas* spp. (RSB5.4 [P1] and RSB5.11 [P3]) persisted, but declined over time, whereas the *Stenotrophomonas* THA2.2 strain that was already commonly found on *X. laevis* (P2) remained stable. The tendency for some probiotics to persist but decrease in ASV sequence count in this experiment generally agrees with results in other amphibian multi-week probiotic experiments where the probiotics similarly declined over time (21, 23). Other work using longer-term multi-month probiotic experiments have also shown overall persistence with a gradual decrease of the probiotic (20), indicating a possible general phenomenon associated with skin probiotic application.

The probiotics used in this study significantly impacted the ASV sequence count and relative abundance of anti-*Bd* bacterial ASVs on *X. laevis* skin. We found that the *Stenotrophomonas* THA2.2 (P2) and *Pseudomonas* RSB5.11 (P3) treatments produced a significant reduction in anti-*Bd* bacteria ASV richness over time when excluding these added probiotics. This suggests that these probiotics were replacing at least one of the pre-treatment anti-*Bd* bacteria, perhaps through some form of competitive exclusion. Interestingly, relative abundance of anti-*Bd* bacteria ASVs (with the probiotics’ excluded) indicated a significant decline in the anti-*Bd* bacterial ASV relative abundance already present prior to probiotic inoculation. Together, this indicates that adding anti-*Bd* probiotics can reduce the richness and relative abundance of some anti-*Bd* bacteria ASVs present. This may be due to an anti-*Bd* bacterial niche in the community that constrains the total number and relative abundance of individuals in that niche. This would be in agreement with previous work that found the diversity of anti-*Bd* bacteria on amphibian skin to be constrained evolutionarily by the host species (75), suggesting that each host species hosts a particular niche for inhibitory taxa. One limitation of this finding is that there may be additional anti-*Bd* bacteria present that were not contained in the strict *Bd-*inhibitory database (76). However, the strict database used was sourced from a larger initial database representing >7,300 bacterial isolates from >180 frog and salamander species from diverse geographies, which we believe is useful to make reasonable estimates on the number of anti-*Bd* bacteria and their relative abundances (76).

Probiotic application caused significant shifts to bacterial community composition (i.e., beta diversity) across all treatments (Fig. 4). While there were subtle differences between some treatments pre-probiotic exposure for presence-absence measure of composition (Jaccard), no differences were observed in the abundance-weighted measure (Bray-Curtis). These differences were dramatically increased after probiotic exposure at week 1, with the presence-absence measure exhibiting significant pairwise comparisons between all treatments, and most treatments differing in abundance-weighted beta diversity. These differences declined at week 2, with some differences still detected through week 3 for presence-absence beta diversity, but not abundance-weighted diversity nor beta dispersion. These results indicate that even with subtle differences in beta-diversity pre-exposure, probiotic applications induced significant, but temporary shifts in the community. Significant treatment differences were dramatically increased at week 1 and then returned to a similar stability state at weeks 2 and 3, with some minimal, persisting effects. When examined, other studies have similarly observed large shifts in community composition following initial probiotic application that dissipate relatively quickly (20, 25). However, most probiotic studies in wildlife have not examined how probiotic application impacts the resident microbiome (1), and we suggest that this should be studied when possible. Such findings will provide critical information about symbiotic microbial community stability and the likelihood for alternative stable states (76).

All of the immune genes examined in this work using RT-qPCR, with the exception of *FOXP3,* are expressed not only by leukocytes but also by other cell types, keratinocytes notably amongst them. Of all examined genes, only *FOXP3* and *CSF1* were seen to have robust independence in their expression profiles. By contrast, the gene expression of all other RT-qPCR examined immune genes was all highly correlated. We selected *TNFA* and *IL10* as hallmark pro- and anti-inflammatory cytokines, respectively (62, 66). Possibly, the observed strong correlation between these two genes may reflect a compensatory mechanism whereby the expression of one of these cytokines is meant to counteract the effects of the other. Furthermore, we observed that *Pseudomonas* RSB5.4 (P1) mediated higher *TNFA* expression compared to the cocktail in RT-qPCR data. Future work will discern whether the gene expression profiles reported here represent skin-resident leukocyte and/or keratinocyte gene expression and the mechanisms by which these gene products participate in frog skin-microbiome interactions.

*FOXP3* is a key regulatory transcription factor and marker of T regulatory cells (67), which are critical to immune suppression and tolerogenic responses. We found that probiotic application with *Pseudomonas* RSB5.4 (P1) caused downregulation of *FOXP3* expression. This is akin to the effect of *Pseudomonas aeruginosa* in murine probiotic studies, where a *P. aeruginosa* only treatment caused downregulation of *FOXP3* as compared to a combined *P. aeruginosa* and *Lactobacillus rhamnosus* GG treatment (77). Application of this bacterium may thus have disrupted tolerogenic (Treg) responses in favor of immune activation. This is reflected in the elevated immune gene expression seen in this treatment group by RNAseq analysis. *Pseudomonas* RSB5.4 (P1) is likely a new species that has high 16S ribosomal RNA gene sequence identity (99.77%; NCBI BLAST) to *Pseudomonas baetica*; a *Pseudomonas* species with known pathogenic phenotypes (78), while the whole genome has high homology to *Pseudomonas koreensis,* known for its anti-fungal properties (79). Possibly, either through some pathogenic determinants and/or by alarming the skin immune system, *Pseudomonas* RSB5.4 (P1) could have elicited the frog skin immune response away from Treg-mediated tolerance. Interestingly, host transcriptomic analysis revealed that the *Pseudomonas* RSB5.4 (P1) had a relatively larger number of DEGs than the other treatments, highlighting potentially larger effects of *Pseudomonas* RSB5.4 (P1) on *X. laevis* physiology. The skin expression of *FOXP3* as well as the other immune genes examined here could also reflect changes in the gene expression within the same cell subsets, changes in the skin cell composition, such as numbers of Tregs and other leukocytes, or some combination of both.

We were surprised to find differentially expressed antiviral genes following *Pseudomonas* RSB5.4 (P1) treatments, as seen in the RNAseq data. These changes may reflect changes in the skin leukocyte composition or some effort on the part of the microbe to shift the skin-resident immunity away from antimicrobial. Conversely, the observed increase in expression of genes associated with complement pathways and recognition of bacterial components is intuitive.

It is notable that the RNA-seq analysis did not confirm significant differential expression of those genes that we examined by RT-qPCR, although some RT-qPCR examined genes had constitutive expression in the RNA-seq analysis. Complete overlap of the same expression results between RT-qPCR and RNA-seq is not generally expected (80). In the case of *FOXP3*, low abundance levels due to its initiation/activation role in signal cascades and cell differentiation likely explain the lack of significant differential expression seen in the RNA-seq data, as changes in expression of high-abundance transcripts are more detectable and less error-prone. Nevertheless, detectable up-regulation of genes like *IL17C (Pseudomonas tolaasii RSB5.11* [P3] vs control [C] at week 1; Supplementary File 5, Sheet D), which may be induced by FOXP3^+^ Tregs (81), are likely amplified indicators of initial subtle changes in *FOXP3* expression as detected by RT-qPCR.

In our transcriptomics, we also identified GO terms that appear to be hallmarks of probiotic colonization, at least for the bacteria we studied here. These include definitive hemopoiesis (generation of cells that develop into all mature blood and immune cells) and differentiation of monocytes and myeloid cells (a group of immune cells having major roles in innate immunity) at week 1 and opsonization (a process of coating cells or pathogens for the immune system to destroy) at week 3. As GO terms are generated from multi-species databases, it remains unclear if these terms are truly indicative of alteration in *X. laevis* immune responses, or more general associations of probiotic colonization.

It has generally been hypothesized that *Bd* inhibition by bacterial commensals may at least in part be due to their modulation of skin-resident immunity, among other hypotheses such as direct competition (11, 20, 21, 23, 24). Our study is the first to have systematically quantified the impact of probiotic application on the immune system in amphibians, showing that indeed the immune system is affected by probiotic application. Our findings are in line with observations in probiotic research in aquaculture, where immune genes affected by probiotic application are also observed (82). Host responses to microbial colonization that maintain symbiotic homeostasis, expectantly, appear to be more subtle than those responses caused by *Bd* infection (18, 32, 83).

Most immune responses in this study, as measured by skin transcriptomics and RT-qPCR, were probiotic treatment specific, providing a starting point to ask important questions about immune gene variability in the context of *Bd* infection. For future experiments with *Bd*, we speculate that *Pseudomonas* RSB5.4 (P1) and *Stenotrophomonas* THA2.2 (P2) would be the most informative candidates since they showed contrasting patterns of probiotic persistence and immune system cross-talk. *Stenotrophomonas* persisted longest in monoculture, was commonly detected on these frogs without probiotic application, and had no significant association with the top 32 differential gene expression modules, and thus had limited impacts on immune changes. This would be important as a potential probiotic that could persist with anti-*Bd* activity without eliciting a strong immune response. Inversely, *Pseudomonas* RSB5.4 showed a gradual decrease in abundance, effects on *FOXP3* expression, and elicited a higher number of DEGs. *Pseudomonas* more broadly showed positive correlations with gene expression module ME7, a module with a high proportion of host immune system process GO terms. This probiotic may therefore have anti-*Bd* potential indirectly by modulation of the host immune response. In contexts such as anti-*Bd* microbial prophylaxis, it is important to consider that an alteration of the host amphibian skin microbiome may alter those animals’ capacity to mount an appropriate and controlled immune response (17, 84). Alteration to the microbiome by probiotic application may instead augment the pathogenic effects of *Bd*, such as inflammation-associated pathology, thus increasing the likelihood of host mortality (18, 25, 83). How probiotic application with bacteria known to also interact with the immune system would affect chytridiomycosis outcomes is presently unknown. Better understanding of the absolute abundance of bacteria present in the skin microbiome would also be important in future work examining host immune responses and chytridiomycosis outcomes (85). This might be accomplished using new and developing spike-in control procedures for 16S rRNA gene sequencing (86, 87). Having a clearer understanding of these mechanisms through future work with probiotics having contrasting mechanisms of action such as these will result in predictive and exploitable strategies for probiotic therapies of disease modulation generally, and better inform risks associated with such therapies.

Our study is the first in amphibians to systematically demonstrate the impact of probiotic application on overall immune system function, supplementing ongoing investigation on the amphibian immune-microbiome interface (16, 31, 32, 71) and amphibian evolutionary ecology more broadly (88). While many aspects of microbial community structure and innate immunity mechanisms are phylogenetically conserved (75, 89), understanding mechanisms in amphibian microbial-immune cross-talk is in its infancy with important implications for disease ecology and organismal evolutionary ecology including speciation and extinction. A recent theory proposed by Woodhams et al. (7) coined as the “adaptive microbiome hypothesis” posits that microbial-immune interactions are a process of disease resilience, whereby competitive microbial interactions interact with differential host immunity to select for functions that increase host fitness. An alternative explanation to this adaptive-microbiome hypothesis is a “constrained” microbiome hypothesis, where specific intrinsic factors such as evolutionarily constrained host-associated microbial composition differentially restrict the possibility space of immune responses in a manner that is causally independent from adaptive-selective mechanisms (90). While our study provides support for both hypotheses, context dependency (91) and future amphibian immune-microbiome studies will help resolve their potential role in wildlife disease outcomes and eco-evolutionary processes.

## Data Availability

Genomic data of the probiotic strains used in this study are deposited as BioProject numbers PRJNA1083098 (RSB 5.4), PRJNA1083108 (RSB 5.11), and PRJNA1082356 (THA 2.2) in the NCBI Sequence Read Archive (SRA). All transcriptomic and 16S rRNA gene sequencing data are deposited as BioProject number PRJNA1092815 in the NCBI Sequence Read Archive (SRA). All supplementary files referenced are included as Supplementary Files 1 and 2 in FigShare (https://figshare.com/articles/journal_contribution/Supplementary_File_1_xlsx/27984488?file=51037448 and https://figshare.com/articles/journal_contribution/Supplementary_File_2_fasta/27984491?file=51037451). Code files used in the analysis of the data have been deposited as Jupyter notebooks in Github at https://github.com/kvasir7/Smithsonian_Xenopus_Probiotic_Project and also as raw code at https://github.com/ogosborne/Xenopus_probiotics_RNAseq. The database with strict curation of *Bd* inhibitory bacteria isolates used in this study is publicly available at https://github.com/AmphiBac/AmphiBac-Database.

## SUPPLEMENTAL MATERIAL

Supplemental material iai.00569-24-s0001.pdf Fig. S1 to S7; Tables S1 to S10.

ASM does not own the copyrights to Supplemental Material that may be linked to, or accessed through, an article. The authors have granted ASM a non-exclusive, world-wide license to publish the Supplemental Material files. Please contact the corresponding author directly for reuse.

## References

[1] Bornbusch SL, Power ML, Schulkin J, Drea CM, Maslanka MT, Muletz-Wolz CR. 2024. Integrating microbiome science and evolutionary medicine into animal health and conservation. Biol Rev Camb Philos Soc 99:458–477. doi:10.1111/brv.13030 37956701

[2] Garcias-Bonet N, Roik A, Tierney B, García FC, Villela HDM, Dungan AM, Quigley KM, Sweet M, Berg G, Gram L, Bourne DG, Ushijima B, Sogin M, Hoj L, Duarte G, Hirt H, Smalla K, Rosado AS, Carvalho S, Thurber RV, Ziegler M, Mason CE, van Oppen MJH, Voolstra CR, Peixoto RS. 2024. Horizon scanning the application of probiotics for wildlife. Trends Microbiol 32:252–269. doi:10.1016/j.tim.2023.08.012 37758552

[3] Huang S, Jiang S, Huo D, Allaband C, Estaki M, Cantu V, Belda-Ferre P, Vázquez-Baeza Y, Zhu Q, Ma C, Li C, Zarrinpar A, Liu Y-Y, Knight R, Zhang J. 2021. Candidate probiotic Lactiplantibacillus plantarum HNU082 rapidly and convergently evolves within human, mice, and zebrafish gut but differentially influences the resident microbiome. Microbiome 9:151. doi:10.1186/s40168-021-01102-0 34193290 PMC8247228

[4] Trush EA, Poluektova EA, Beniashvilli AG, Shifrin OS, Poluektov YM, Ivashkin VT. 2020. The evolution of human probiotics: challenges and prospects. Probiotics Antimicrob Proteins 12:1291–1299. doi:10.1007/s12602-019-09628-4 31907861

[5] Honda K, Littman DR. 2016. The microbiota in adaptive immune homeostasis and disease. Nature New Biol 535:75–84. doi:10.1038/nature18848 27383982

[6] Kau AL, Ahern PP, Griffin NW, Goodman AL, Gordon JI. 2011. Human nutrition, the gut microbiome and the immune system. Nature New Biol 474:327–336. doi:10.1038/nature10213 PMC3298082 21677749

[7] Woodhams DC, McCartney J, Walke JB, Whetstone R. 2023. The adaptive microbiome hypothesis and immune interactions in amphibian mucus. Dev Comp Immunol 145:104690. doi:10.1016/j.dci.2023.104690 37001710 PMC10249470

[8] Bosch TCG. 2013. Cnidarian-microbe interactions and the origin of innate immunity in metazoans. Annu Rev Microbiol 67:499–518. doi:10.1146/annurev-micro-092412-155626 23808329

[9] Lee YK, Mazmanian SK. 2010. Has the microbiota played a critical role in the evolution of the adaptive immune system? Science 330:1768–1773. doi:10.1126/science.1195568 21205662 PMC3159383

[10] Luedtke JA, Chanson J, Neam K, Hobin L, Maciel AO, Catenazzi A, Borzée A, Hamidy A, Aowphol A, Jean A, et al.. 2023. Ongoing declines for the world’s amphibians in the face of emerging threats. Nature New Biol 622:308–314. doi:10.1038/s41586-023-06578-4 PMC10567568 37794184

[11] Bletz MC, Loudon AH, Becker MH, Bell SC, Woodhams DC, Minbiole KPC, Harris RN. 2013. Mitigating amphibian chytridiomycosis with bioaugmentation: characteristics of effective probiotics and strategies for their selection and use. Ecol Lett 16:807–820. doi:10.1111/ele.12099 23452227

[12] Rollins-Smith LA. 2020. Global amphibian declines, disease, and the ongoing battle between Batrachochytrium fungi and the immune system. Herpetologica 76:178. doi:10.1655/0018-0831-76.2.178

[13] Lips KR. 2016. Overview of chytrid emergence and impacts on amphibians. Philos Trans R Soc Lond B Biol Sci 371:20150465. doi:10.1098/rstb.2015.0465 28080989 PMC5095542

[14] Van Rooij P, Martel A, Haesebrouck F, Pasmans F. 2015. Amphibian chytridiomycosis: a review with focus on fungus-host interactions. Vet Res 46:137. doi:10.1186/s13567-015-0266-0 26607488 PMC4660679

[15] Siomko SA, Greenspan SE, Barnett KM, Neely WJ, Chtarbanova S, Woodhams DC, McMahon TA, Becker CG. 2023. Selection of an anti-pathogen skin microbiome following prophylaxis treatment in an amphibian model system. Philos Trans R Soc Lond B Biol Sci 378:20220126. doi:10.1098/rstb.2022.0126 37305917 PMC10258671

[16] Woodhams DC, Rollins-Smith LA, Reinert LK, Lam BA, Harris RN, Briggs CJ, Vredenburg VT, Patel BT, Caprioli RM, Chaurand P, Hunziker P, Bigler L. 2020. Probiotics modulate a novel amphibian skin defense peptide that is antifungal and facilitates growth of antifungal bacteria. Microb Ecol 79:192–202. doi:10.1007/s00248-019-01385-9 31093727

[17] Muletz-Wolz CR, Fleischer RC, Lips KR. 2019. Fungal disease and temperature alter skin microbiome structure in an experimental salamander system. Mol Ecol 28:2917–2931. doi:10.1111/mec.15122 31066947

[18] Ellison A, Zamudio K, Lips K, Muletz-Wolz C. 2020. Temperature-mediated shifts in salamander transcriptomic responses to the amphibian-killing fungus. Mol Ecol 29:325–343. doi:10.1111/mec.15327 31820839

[19] McKenzie VJ, Kueneman JG, Harris RN. 2018. Probiotics as a tool for disease mitigation in wildlife: insights from food production and medicine. Ann N Y Acad Sci 1429:18–30. doi:10.1111/nyas.13617 29479716

[20] Kueneman JG, Woodhams DC, Harris R, Archer HM, Knight R, McKenzie VJ. 2016. Probiotic treatment restores protection against lethal fungal infection lost during amphibian captivity. Proc R Soc B 283:20161553. doi:10.1098/rspb.2016.1553 PMC5046908 27655769

[21] Muletz CR, Myers JM, Domangue RJ, Herrick JB, Harris RN. 2012. Soil bioaugmentation with amphibian cutaneous bacteria protects amphibian hosts from infection by Batrachochytrium dendrobatidis. Biol Conserv 152:119–126. doi:10.1016/j.biocon.2012.03.022

[22] Robak MJ, Richards-Zawacki CL. 2018. Temperature-dependent effects of cutaneous bacteria on a frog’s tolerance of fungal infection. Front Microbiol 9:410. doi:10.3389/fmicb.2018.00410 29563909 PMC5845872

[23] Madison JD, Ouellette SP, Schmidt EL, Kerby JL. 2019. Serratia marcescens shapes cutaneous bacterial communities and influences survival of an amphibian host. Proc Biol Sci 286:20191833. doi:10.1098/rspb.2019.1833 31662077 PMC6842860

[24] Woodhams DC, Geiger CC, Reinert LK, Rollins-Smith LA, Lam B, Harris RN, Briggs CJ, Vredenburg VT, Voyles J. 2012. Treatment of amphibians infected with chytrid fungus: learning from failed trials with itraconazole, antimicrobial peptides, bacteria, and heat therapy. Dis Aquat Organ 98:11–25. doi:10.3354/dao02429 22422126

[25] Becker MH, Brophy JAN, Barrett K, Bronikowski E, Evans M, Glassey E, Kaganer AW, Klocke B, Lassiter E, Meyer AJ, Muletz-Wolz CR, Fleischer RC, Voigt CA, Gratwicke B. 2021. Genetically modifying skin microbe to produce violacein and augmenting microbiome did not defend Panamanian golden frogs from disease. ISME Commun 1:57. doi:10.1038/s43705-021-00044-w 37938636 PMC9723765

[26] Cheng TL, Mayberry H, McGuire LP, Hoyt JR, Langwig KE, Nguyen H, Parise KL, Foster JT, Willis CKR, Kilpatrick AM, Frick WF. 2017. Efficacy of a probiotic bacterium to treat bats affected by the disease white‐nose syndrome. Journal of Applied Ecology 54:701–708. doi:10.1111/1365-2664.12757

[27] Yong E. 2016. I Contain Multitudes: The Microbes within Us and a Grander View of Life. HarperCollins Publishers, New York, NY.

[28] Muletz-Wolz CR, DiRenzo GV, Yarwood SA, Campbell Grant EH, Fleischer RC, Lips KR. 2017. Antifungal bacteria on woodland salamanders exhibit high taxonomic diversity and geographic variability. Appl Environ Microbiol 83:e00186–17. doi:10.1128/AEM.00186-17 28213545 PMC5394319

[29] Andersen KG, Nissen JK, Betz AG. 2012. Comparative genomics reveals key gain-of-function events in Foxp3 during regulatory T cell evolution. Front Immunol 3:113. doi:10.3389/fimmu.2012.00113 22590469 PMC3349156

[30] Muletz Wolz CR, Yarwood SA, Campbell Grant EH, Fleischer RC, Lips KR. 2018. Effects of host species and environment on the skin microbiome of Plethodontid salamanders. J Anim Ecol 87:341–353. doi:10.1111/1365-2656.12726 28682480

[31] Jiménez RR, Carfagno A, Linhoff L, Gratwicke B, Woodhams DC, Chafran LS, Bletz MC, Bishop B, Muletz-Wolz CR. 2022. Inhibitory bacterial diversity and mucosome function differentiate susceptibility of Appalachian salamanders to chytrid fungal infection. Appl Environ Microbiol 88:e01818–21. doi:10.1128/aem.01818-21 35348389 PMC9040618

[32] Hauser KA, Garvey CN, Crow RS, Hossainey MRH, Howard DT, Ranganathan N, Gentry LK, Yaparla A, Kalia N, Zelle M, Jones EJ, Duttargi AN, Rollins-Smith LA, Muletz-Wolz CR, Grayfer L. 2024. Amphibian mast cells serve as barriers to chytrid fungus infections. Elife 12:RP92168. doi:10.7554/eLife.92168 39082933 PMC11290838

[33] Kolmogorov M, Yuan J, Lin Y, Pevzner PA. 2019. Assembly of long, error-prone reads using repeat graphs. Nat Biotechnol 37:540–546. doi:10.1038/s41587-019-0072-8 30936562

[34] Meier-Kolthoff JP, Göker M. 2019. TYGS is an automated high-throughput platform for state-of-the-art genome-based taxonomy. Nat Commun 10:2182. doi:10.1038/s41467-019-10210-3 31097708 PMC6522516

[35] Alanjary M, Steinke K, Ziemert N. 2019. AutoMLST: an automated web server for generating multi-locus species trees highlighting natural product potential. Nucleic Acids Res 47:W276–W282. doi:10.1093/nar/gkz282 30997504 PMC6602446

[36] Muletz-Wolz CR, Almario JG, Barnett SE, DiRenzo GV, Martel A, Pasmans F, Zamudio KR, Toledo LF, Lips KR. 2017. Inhibition of fungal pathogens across genotypes and temperatures by amphibian skin bacteria. Front Microbiol 8:1551. doi:10.3389/fmicb.2017.01551 28871241 PMC5566582

[37] Kanyó I, Molnár LV. 2016. Procaryotic species and subspecies delineation using average nucleotide identity and gene order conservation. Gene Rep 5:75–82. doi:10.1016/j.genrep.2016.09.004

[38] Bhattarai K. 2023. Chemical Investigation of Chytrid Fungi and Amphibian Skin Microbiome Bacteria PhD thesis, Universität Tübingen, Tübingen, Germany

[39] McKenzie VJ, Bowers RM, Fierer N, Knight R, Lauber CL. 2012. Co-habiting amphibian species harbor unique skin bacterial communities in wild populations. ISME J 6:588–596. doi:10.1038/ismej.2011.129 21955991 PMC3280140

[40] Kueneman JG, Parfrey LW, Woodhams DC, Archer HM, Knight R, McKenzie VJ. 2014. The amphibian skin-associated microbiome across species, space and life history stages. Mol Ecol 23:1238–1250. doi:10.1111/mec.12510 24171949

[41] Bornbusch SL, Shinnerl HE, Gentry L, Keady MM, Glick V, Muletz-Wolz CR, Power ML. 2024. Local environment shapes milk microbiomes while evolutionary history constrains milk macronutrients in captive cercopithecine primates. Environ Microbiol 26:e16664. doi:10.1111/1462-2920.16664 38830671

[42] Livak KJ, Schmittgen TD. 2001. Analysis of relative gene expression data using real-time quantitative PCR and the 2^−ΔΔCT^ method. Methods 25:402–408. doi:10.1006/meth.2001.1262 11846609

[43] Callahan BJ, McMurdie PJ, Rosen MJ, Han AW, Johnson AJA, Holmes SP. 2016. DADA2: High-resolution sample inference from Illumina amplicon data. Nat Methods 13:581–583. doi:10.1038/nmeth.3869 27214047 PMC4927377

[44] Davis NM, Proctor DM, Holmes SP, Relman DA, Callahan BJ. 2018. Simple statistical identification and removal of contaminant sequences in marker-gene and metagenomics data. Microbiome 6:226. doi:10.1186/s40168-018-0605-2 30558668 PMC6298009

[45] McMurdie PJ, Holmes S. 2013. Phyloseq: an R package for reproducible interactive analysis and graphics of microbiome census data. PLoS One 8:e61217. doi:10.1371/journal.pone.0061217 23630581 PMC3632530

[46] Oksanen J, Simpson GL, Blanchet FG, Kindt R, Legendre P, Minchin PR, Weedon J. 2022. Vegan: community ecology package, 2.6-2. R Foundation for Statistical Computing, Vienna (Austria).

[47] Wickham H. 2016. Data analysis, p 189–201. Springer International Publishing.

[48] Woodhams DC, Alford RA, Antwis RE, Archer H, Becker MH, Belden LK, Bell SC, Bletz M, Daskin JH, Davis LR, et al.. 2015. Antifungal isolates database of amphibian skin‐associated bacteria and function against emerging fungal pathogens: Ecological Archives E096‐059. Ecology 96:595–595. doi:10.1890/14-1837.1

[49] Ewels PA, Peltzer A, Fillinger S, Patel H, Alneberg J, Wilm A, Garcia MU, Di Tommaso P, Nahnsen S. 2020. The nf-core framework for community-curated bioinformatics pipelines. Nat Biotechnol 38:276–278. doi:10.1038/s41587-020-0439-x 32055031

[50] Di Tommaso P, Chatzou M, Floden EW, Barja PP, Palumbo E, Notredame C. 2017. Nextflow enables reproducible computational workflows. Nat Biotechnol 35:316–319. doi:10.1038/nbt.3820 28398311

[51] Dobin A, Davis CA, Schlesinger F, Drenkow J, Zaleski C, Jha S, Batut P, Chaisson M, Gingeras TR. 2013. STAR: ultrafast universal RNA-seq aligner. Bioinformatics 29:15–21. doi:10.1093/bioinformatics/bts635 23104886 PMC3530905

[52] Patro R, Duggal G, Love MI, Irizarry RA, Kingsford C. 2017. Salmon provides fast and bias-aware quantification of transcript expression. Nat Methods 14:417–419. doi:10.1038/nmeth.4197 28263959 PMC5600148

[53] Session AM, Uno Y, Kwon T, Chapman JA, Toyoda A, Takahashi S, Fukui A, Hikosaka A, Suzuki A, Kondo M, et al.. 2016. Genome evolution in the allotetraploid frog Xenopus laevis. Nature New Biol 538:336–343. doi:10.1038/nature19840 PMC5313049 27762356

[54] Pertea G, Pertea M. 2020. GFF utilities: GffRead and GffCompare. F1000Res 9:ISCB Comm J-304. doi:10.12688/f1000research.23297.2 PMC7222033 32489650

[55] Cantalapiedra CP, Hernández-Plaza A, Letunic I, Bork P, Huerta-Cepas J. 2021. eggNOG-mapper v2: functional annotation, orthology assignments, and domain prediction at the metagenomic scale. Mol Biol Evol 38:5825–5829. doi:10.1093/molbev/msab293 34597405 PMC8662613

[56] Love MI, Huber W, Anders S. 2014. Moderated estimation of fold change and dispersion for RNA-seq data with DESeq2. Genome Biol 15:550. doi:10.1186/s13059-014-0550-8 25516281 PMC4302049

[57] Conway JR, Lex A, Gehlenborg N. 2017. UpSetR: an R package for the visualization of intersecting sets and their properties. Bioinformatics 33:2938–2940. doi:10.1093/bioinformatics/btx364 28645171 PMC5870712

[58] Alexa A, Rahnenfuhrer J. 2018. topGO: enrichment analysis for gene ontology. In R package version 2.46.0

[59] Zhang B, Horvath S. 2005. A general framework for weighted gene co-expression network analysis. Stat Appl Genet Mol Biol 4:Article17. doi:10.2202/1544-6115.1128 16646834

[60] Langfelder P, Horvath S. 2008. WGCNA: an R package for weighted correlation network analysis. BMC Bioinformatics 9:1–13. doi:10.1186/1471-2105-9-559 19114008 PMC2631488

[61] Bastian M, Heymann S, Jacomy M. 2009. Gephi: an open source software for exploring and manipulating networks. ICWSM 3:361–362. doi:10.1609/icwsm.v3i1.13937

[62] Saraiva M, Vieira P, O’Garra A. 2020. Biology and therapeutic potential of interleukin-10. J Exp Med 217:e20190418. doi:10.1084/jem.20190418 31611251 PMC7037253

[63] Hossainey MRH, Hauser KA, Garvey CN, Kalia N, Garvey JM, Grayfer L. 2023. A perspective into the relationships between amphibian (Xenopus laevis) myeloid cell subsets. Philos Trans R Soc Lond B Biol Sci 378:20220124. doi:10.1098/rstb.2022.0124 37305910 PMC10258660

[64] Tie Y, Tang F, Peng D, Zhang Y, Shi H. 2022. TGF-beta signal transduction: biology, function and therapy for diseases. Mol Biomed 3:45. doi:10.1186/s43556-022-00109-9 36534225 PMC9761655

[65] Hari A, Flach TL, Shi Y, Mydlarski PR. 2010. Toll-like receptors: role in dermatological disease. Mediators Inflamm 2010:437246. doi:10.1155/2010/437246 20847936 PMC2933899

[66] Schottelius AJG, Moldawer LL, Dinarello CA, Asadullah K, Sterry W, Edwards CK III. 2004. Biology of tumor necrosis factor‐α– implications for psoriasis. Exp Dermatol 13:193–222. doi:10.1111/j.0906-6705.2004.00205.x 15086336

[67] Li Z, Li D, Tsun A, Li B. 2015. FOXP3+ regulatory T cells and their functional regulation. Cell Mol Immunol 12:558–565. doi:10.1038/cmi.2015.10 25683611 PMC4579651

[68] Nahm MH, Yu J, Calix JJ, Ganaie F. 2022. Ficolin-2 lectin complement pathway mediates capsule-specific innate immunity against invasive pneumococcal disease. Front Immunol 13:841062. doi:10.3389/fimmu.2022.841062 35418983 PMC8996173

[69] Alexiev A, Chen MY, Korpita T, Weier AM, McKenzie VJ. 2023. Together or alone: evaluating the pathogen inhibition potential of bacterial Cocktails against an amphibian pathogen. Microbiol Spectr 11:e0151822. doi:10.1128/spectrum.01518-22 36719234 PMC10100949

[70] Cortazar-Chinarro M, Richter-Boix A, Rödin-Mörch P, Halvarsson P, Logue JB, Laurila A, Höglund J. 2024. Association between the skin microbiome and MHC class II diversity in an amphibian. Mol Ecol 33:e17198. doi:10.1111/mec.17198 37933583

[71] Davis LR, Bigler L, Woodhams DC. 2017. Developmental trajectories of amphibian microbiota: response to bacterial therapy depends on initial community structure. Environ Microbiol 19:1502–1517. doi:10.1111/1462-2920.13707 28229543

[72] Madison JD. 2021. Stochasticity and randomness in community assembly: real or as-if? mSystems 6:e0093821. doi:10.1128/mSystems.00938-21 34491087 PMC8547434

[73] Jones KR, Belden LK, Hughey MC. 2024. Priority effects alter microbiome composition and increase abundance of probiotic taxa in treefrog tadpoles. Appl Environ Microbiol 90:e0061924. doi:10.1128/aem.00619-24 38757977 PMC11218634

[74] Roughgarden J. 2023. Holobiont evolution: population theory for the hologenome. Am Nat 201:763–778. doi:10.1086/723782 37229712

[75] Osborne OG, Jiménez RR, Byrne AQ, Gratwicke B, Ellison A, Muletz-Wolz CR. 2024. Phylosymbiosis shapes skin bacterial communities and pathogen-protective function in Appalachian salamanders. ISME J 18:wrae104. doi:10.1093/ismejo/wrae104 38861457 PMC11195472

[76] Fujita H, Ushio M, Suzuki K, Abe MS, Yamamichi M, Iwayama K, Canarini A, Hayashi I, Fukushima K, Fukuda S, Kiers ET, Toju H. 2023. Alternative stable states, nonlinear behavior, and predictability of microbiome dynamics. Microbiome 11:63. doi:10.1186/s40168-023-01474-5 36978146 PMC10052866

[77] Khailova L, Baird CH, Rush AA, McNamee EN, Wischmeyer PE. 2013. Lactobacillus rhamnosus GG improves outcome in experimental Pseudomonas aeruginosa pneumonia: potential role of regulatory T cells. Shock 40:496–503. doi:10.1097/SHK.0000000000000066 24240593 PMC5592098

[78] López JR, Diéguez AL, Doce A, De la Roca E, De la Herran R, Navas JI, Toranzo AE, Romalde JL. 2012. Pseudomonas baetica sp. nov., a fish pathogen isolated from wedge sole, Dicologlossa cuneata (Moreau). Int J Syst Evol Microbiol 62:874–882. doi:10.1099/ijs.0.030601-0 21642488

[79] Rafikova GF, Korshunova TY, Minnebaev LF, Chetverikov SP, Loginov ON. 2016. A new bacterial strain, Pseudomonas koreensis IB-4, as A promising agent for plant pathogen biological control. Microbiology (Reading, Engl) 85:333–341. doi:10.1134/S0026261716030115

[80] Everaert C, Luypaert M, Maag JLV, Cheng QX, Dinger ME, Hellemans J, Mestdagh P. 2017. Benchmarking of RNA-sequencing analysis workflows using whole-transcriptome RT-qPCR expression data. Sci Rep 7:1559. doi:10.1038/s41598-017-01617-3 28484260 PMC5431503

[81] Voo KS, Wang Y-H, Santori FR, Boggiano C, Wang Y-H, Arima K, Bover L, Hanabuchi S, Khalili J, Marinova E, Zheng B, Littman DR, Liu Y-J. 2009. Identification of IL-17-producing FOXP3+ regulatory T cells in humans. Proc Natl Acad Sci U S A 106:4793–4798. doi:10.1073/pnas.0900408106 19273860 PMC2653560

[82] Büyükdeveci ME, Cengizler İ, Balcázar JL, Demirkale İ. 2023. Effects of two host-associated probiotics Bacillus mojavensis B191 and Bacillus subtilis MRS11 on growth performance, intestinal morphology, expression of immune-related genes and disease resistance of Nile tilapia (Oreochromis niloticus) against Streptococcus iniae. Dev Comp Immunol 138:104553. 36122732 10.1016/j.dci.2022.104553

[83] Ellison AR, Tunstall T, DiRenzo GV, Hughey MC, Rebollar EA, Belden LK, Harris RN, Ibáñez R, Lips KR, Zamudio KR. 2014. More than skin deep: functional genomic basis for resistance to amphibian chytridiomycosis. Genome Biol Evol 7:286–298. doi:10.1093/gbe/evu285 25539724 PMC4316636

[84] Jani AJ, Briggs CJ. 2014. The pathogen Batrachochytrium dendrobatidis disturbs the frog skin microbiome during a natural epidemic and experimental infection. Proc Natl Acad Sci U S A 111:E5049–58. doi:10.1073/pnas.1412752111 25385615 PMC4250152

[85] Galazzo G, van Best N, Benedikter BJ, Janssen K, Bervoets L, Driessen C, Oomen M, Lucchesi M, van Eijck PH, Becker HEF, Hornef MW, Savelkoul PH, Stassen FRM, Wolffs PF, Penders J. 2020. How to count our microbes? The effect of different quantitative microbiome profiling approaches. Front Cell Infect Microbiol 10:403. doi:10.3389/fcimb.2020.00403 32850498 PMC7426659

[86] Ghotbi M, Stajich JE, Dallas J, Rurik A, Cummins C, Vargas-Gastélum L, Ghotbi M, Spatafora JW, Kelly K, Alexander NR, Moe KC, Syring KC, Shadmani L, Perez-Marron J, Walker DM. 2024. Absolute abundance unveils Basidiobolus as a cross-domain bridge indirectly bolstering gut microbiome homeostasis. Microbiology. doi:10.1101/2024.12.27.630554 PMC12400927 40689579

[87] Rao C, Coyte KZ, Bainter W, Geha RS, Martin CR, Rakoff-Nahoum S. 2021. Multi-kingdom ecological drivers of microbiota assembly in preterm infants. Nature New Biol 591:633–638. doi:10.1038/s41586-021-03241-8 PMC7990694 33627867

[88] Longo AV, Lips KR, Zamudio KR. 2023. Evolutionary ecology of host competence after a chytrid outbreak in a naive amphibian community. Philos Trans R Soc Lond B Biol Sci 378:20220130. doi:10.1098/rstb.2022.0130 37305909 PMC10258670

[89] Pradeu T, Thomma BPHJ, Girardin SE, Lemaitre B. 2024. The conceptual foundations of innate immunity: taking stock 30 years later. Immunity 57:613–631. doi:10.1016/j.immuni.2024.03.007 38599162

[90] Gould SJ. 2002. The structure of evolutionary theory. Harvard University Press, Cambridge, MA.

[91] Madison JD. 2018. Microbial community characterization and restructuring for the amelioration of amphibian chytridiomycosis PhD thesis, University of South Dakota, Vermillion, SD

