# Supplementary material for: Probiotic colonization of Xenopus laevis skin causes short-term changes in skin microbiomes and gene expression
Source: Infect Immun. 2025 Apr 2;93(5):e00569-24. doi: 10.1128/iai.00569-24 (PMC12070741; doi:10.1128/iai.00569-24)
Supplement: Supplemental material — Fig. S1 to S7; Tables S1 to S10. [file iai.00569-24-s0001.pdf]

Supplementary Figures and Tables for:

Probiotic colonization of *Xenopus laevis* skin causes short-term changes in skin microbiomes and gene expression

Joseph D. Madison[1,2,3], Owen G. Osborne[4], Amy Ellison[4], Christina N. Garvey Griffith[5], Lindsey Gentry[1], Harald Gross[6], Brian Gratwicke[7], Leon Grayfer[5], Carly R. Muletz-Wolz[1]

[1]Center for Conservation Genomics, Smithsonian's National Zoo and Conservation Biology Institute, Washington DC, USA
[2]Department of Biology, University of Massachusetts Boston, Boston, MA
[3]Department of Biology, Xavier University of Louisiana, New Orleans, LA
[4]School of Environmental and Natural Sciences, Bangor University, Deiniol Road, Bangor, Gwynedd LL57 2DG, United Kingdom
[5]Department of Biology, George Washington University, Washington DC, USA
[6]Pharmaceutical Institute, Department of Pharmaceutical Biology, University of Tübingen, 72076 Tübingen, Germany
[7]Center for Species Survival, Smithsonian's National Zoo and Conservation Biology Institute, Washington DC, USA

Supplementary Figures

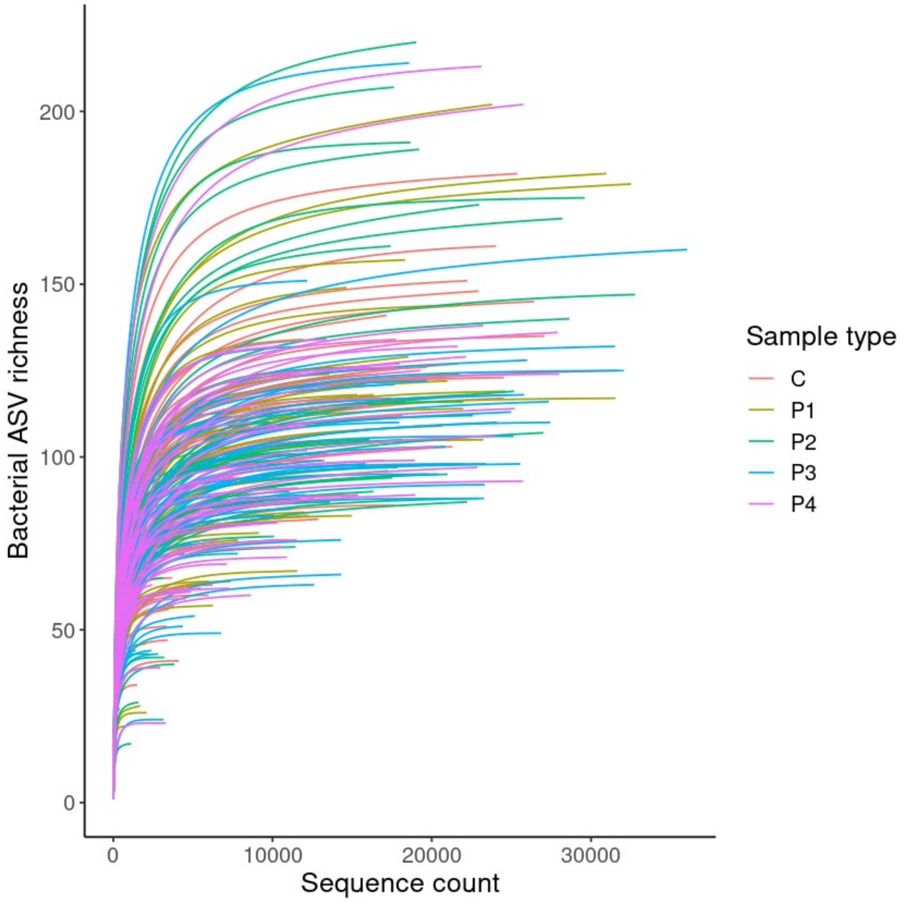

Figure S1. Coverage given (pre-rarefaction) with sequence count plotted against richness. Each line represents a sample. Sample type is given in the legend. C = control, P1 (*Pseudomonas* RSB5.4), P2 (*Stenotrophomonas* THA2.2), P3 (*Pseudomonas* RSB5.11), and P4 (cocktail) are the corresponding probiotic treatments.

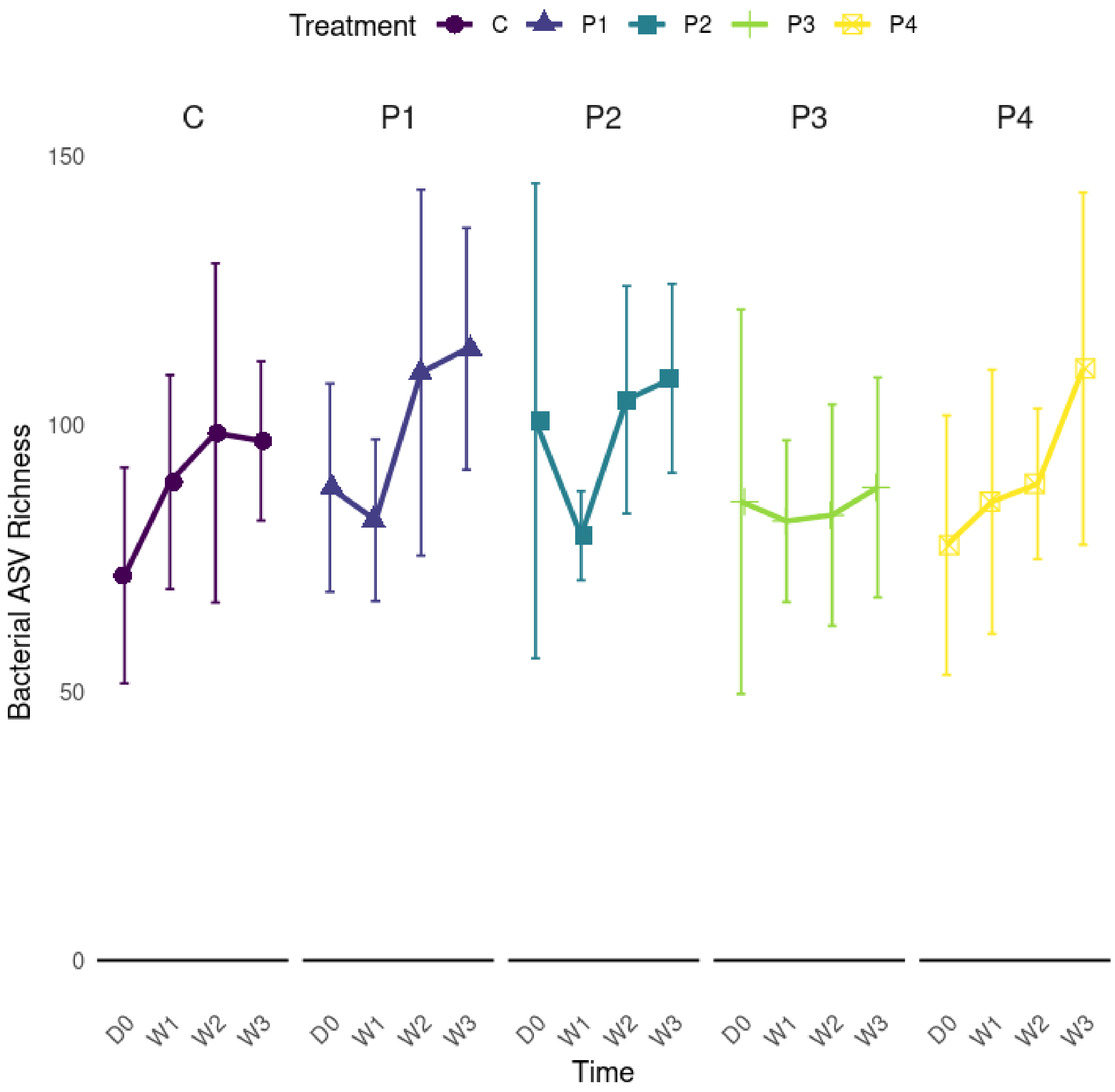

Figure S2. Bar plots of ASV richness (rarefied) by treatment and timepoint. A slight increase in ASV richness was observed over time across treatments and control. Treatments are matched by color/shape and are coded as follows: C = no-probiotic control, P1 = *Pseudomonas* RSB5.4, P2 = *Stenotrophomonas* THA2.2, P3 = *Pseudomonas tolaasii* RSB5.11, P4 = cocktail. Timepoints are given for each treatment and are coded as follows: D0 = day 0, W1 = week 1, W2 = week 2, and W3 = week 3. Error bars at each point represent plus or minus one standard deviation.

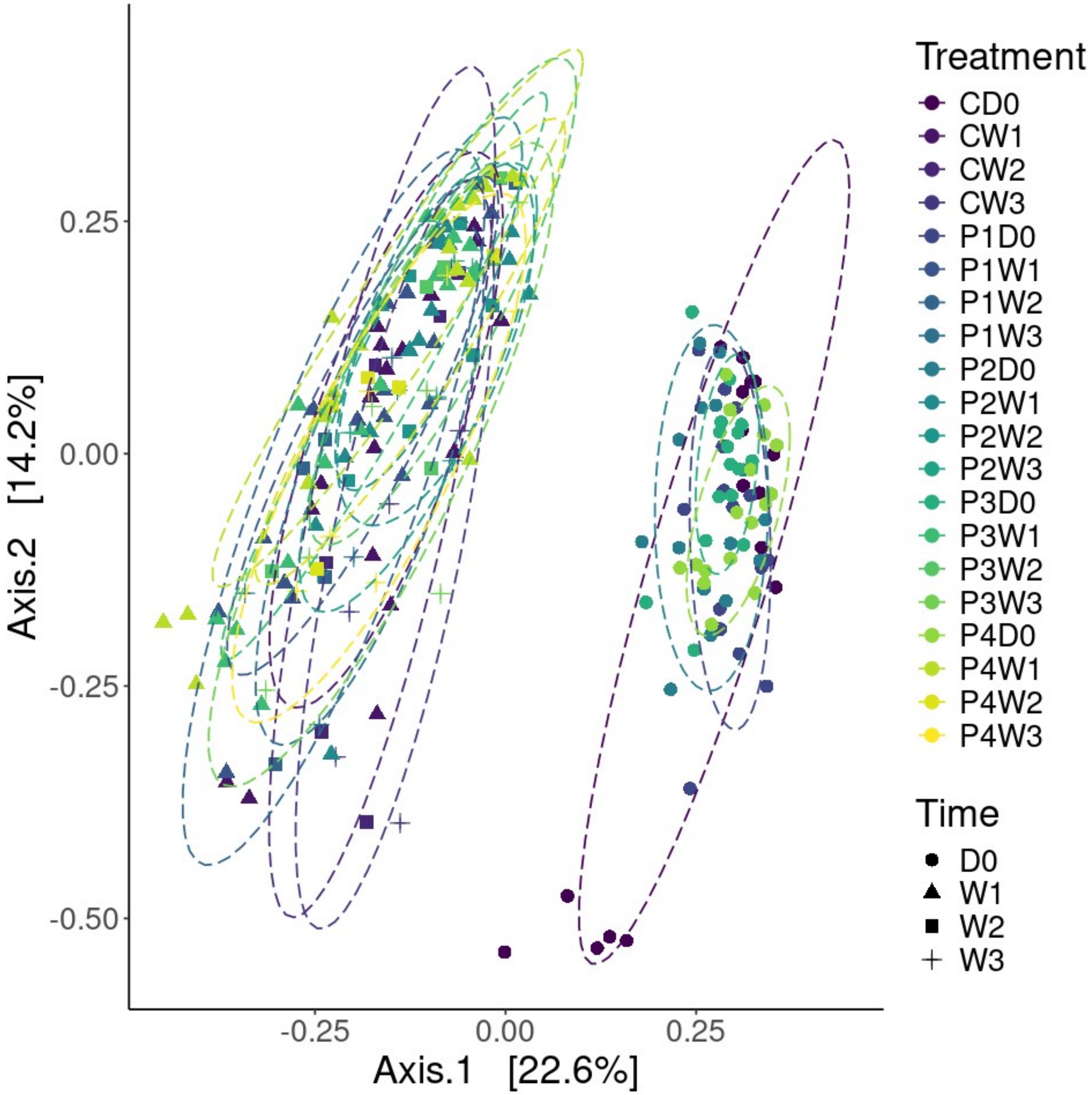

Figure S3. Principal coordinate analyses (axes 1 and 2) of microbial community beta diversity using the Bray-Curtis dissimilarity metric. Treatment group is given by color and time is given by shape, as indicated in the legend. Data ellipses show an 80% confidence and assume a multivariate t-distribution. Treatments are coded as follows: C = no-probiotic control, P1 = *Pseudomonas* RSB5.4, P2 = *Stenotrophomonas* THA2.2, P3 = *Pseudomonas* RSB5.11, P4 = cocktail.

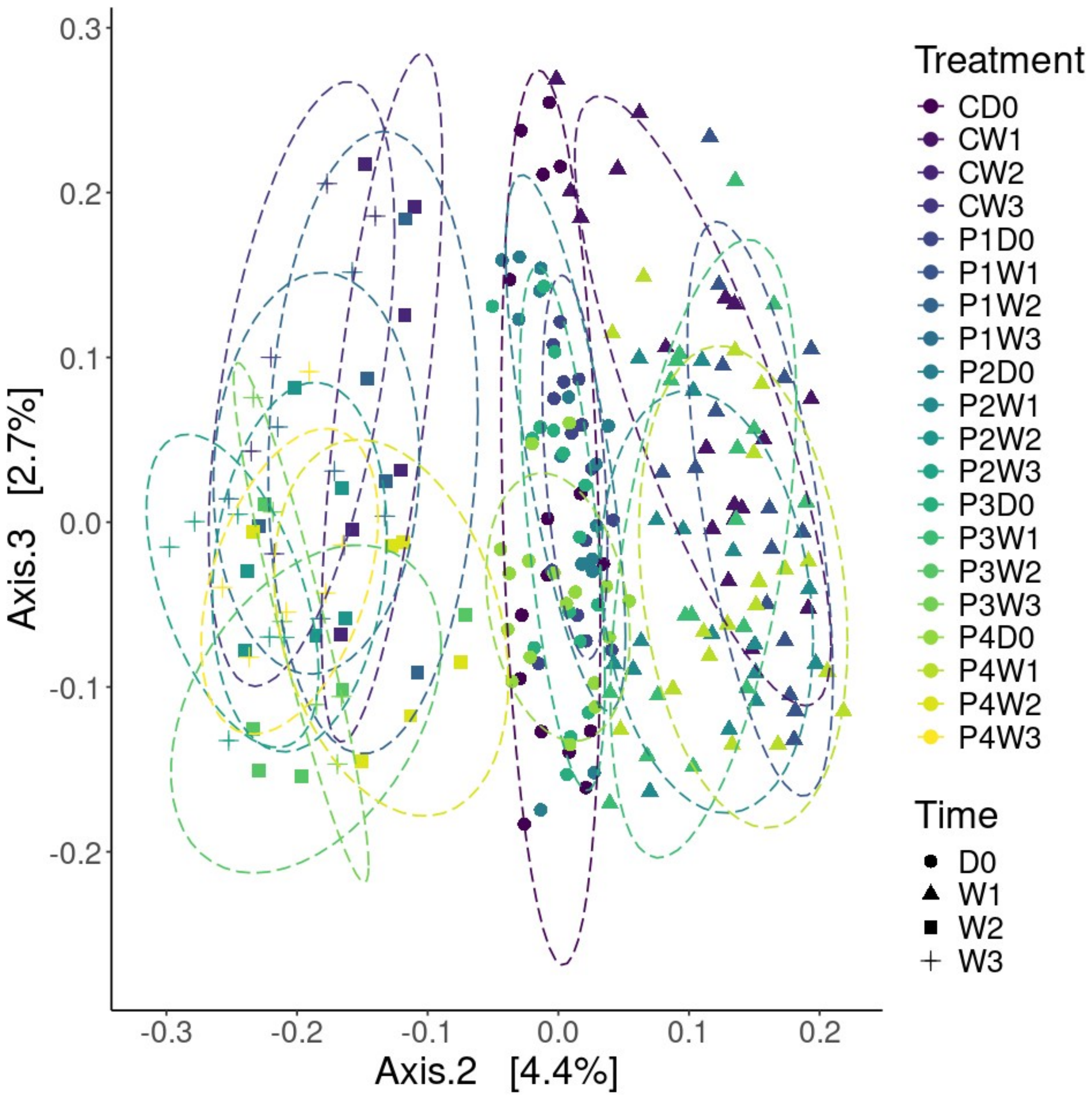

Figure S4. Principal coordinate analyses (axes 2 and 3) of microbial community beta diversity using the Jaccard dissimilarity metric. Treatment group is given by color and time is given by shape, as indicated in the legend. Data ellipses show an 80% confidence and assume a multivariate t-distribution. Treatments are coded as follows: C = no-probiotic control, P1 = *Pseudomonas* RSB5.4, P2 = *Stenotrophomonas* THA2.2, P3 = *Pseudomonas* RSB5.11, P4 = cocktail.

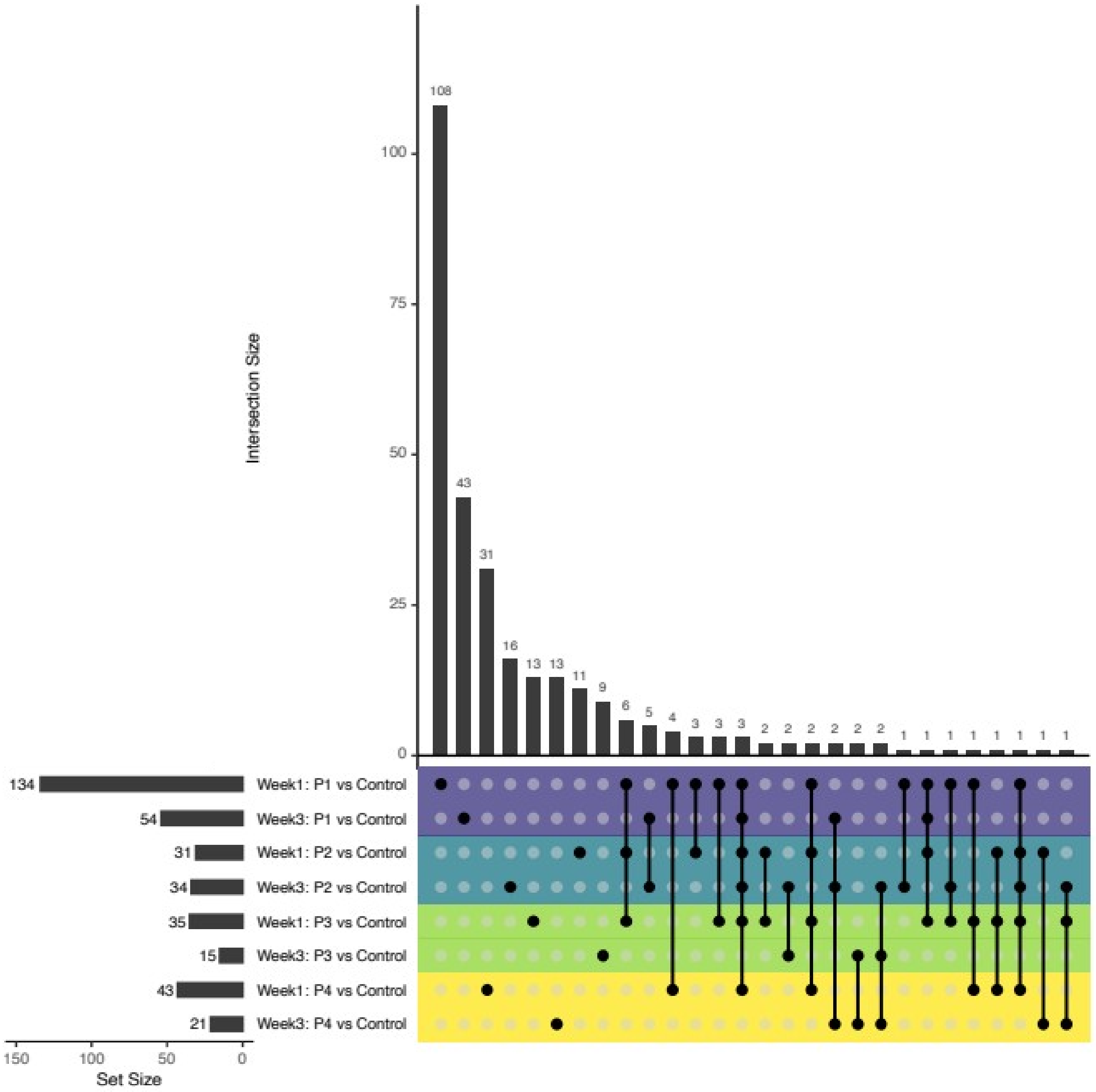

Figure S5. Upset plot showing overlap between each set of all differentially expressed genes. Horizontal bars show the size of each set and vertical bars show the size of each intersection between sets (denoted by black points within the central grid). The rows of the central grid are colored by test treatment as in figures 1–7. Treatments are coded as follows: C = no-probiotic control, P1 = *Pseudomonas* RSB5.4, P2 = *Stenotrophomonas* THA2.2, P3 = *Pseudomonas* RSB5.11, P4 = cocktail.

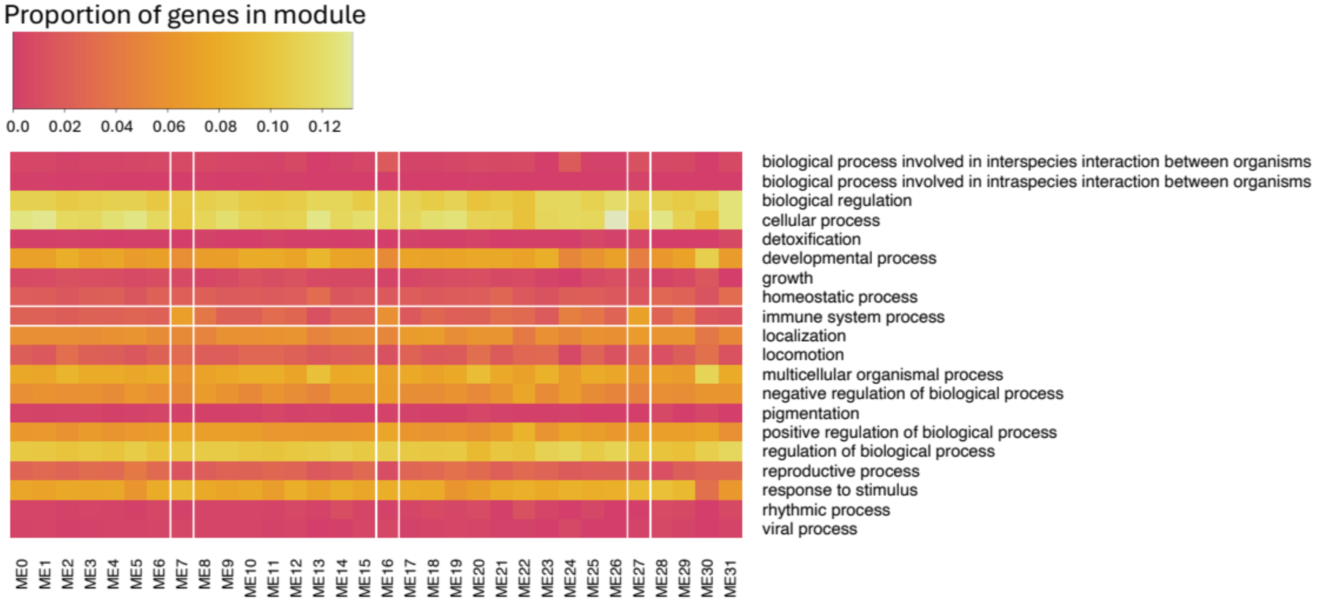

Figure S6. A heatmap showing the proportion of genes in each expression module annotated with each of the level one biological process GO terms. Horizontal lines highlight the "immune systems process" GO term and vertical lines highlight the modules with the highest proportion of genes annotated to it.

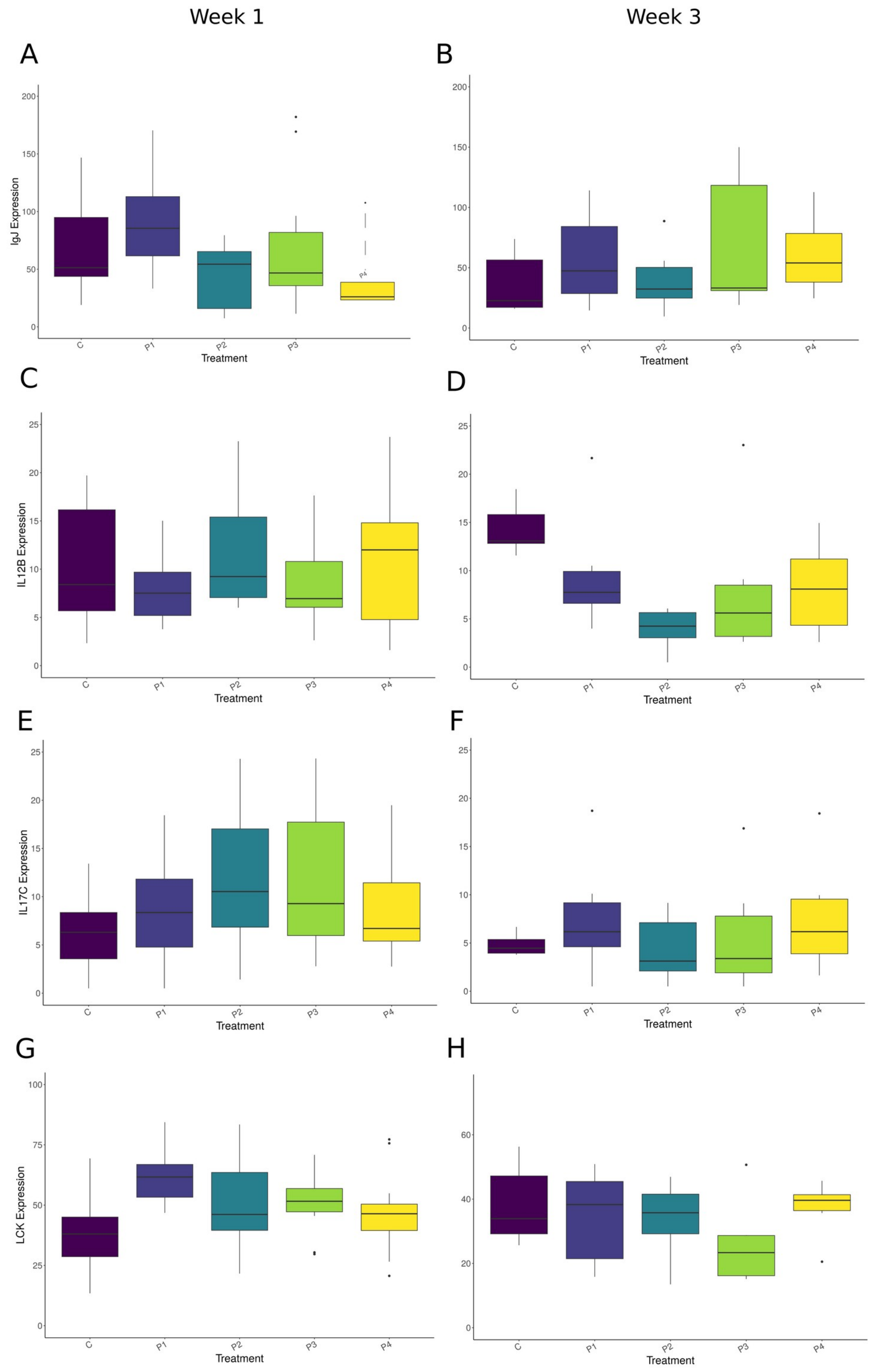

Figure S7. A barplot showing a subset of differentially expressed genes of interest (as compared to control) at weeks 1 and 3 by respective treatment group (P1–P4) and control (C). This subset of genes includes IgJ in (A) and (B), IL12B in (C) and (D), IL17C in (E) and (F), and LCK in (G) and (H). All expression data is normalized by library size. The left column shows week 1 and the right column shows week 3 for the corresponding gene. Treatments are matched by color across panels and are coded as follows: C = no-probiotic control, P1 = *Pseudomonas* RSB5.4, P2 = *Stenotrophomonas* THA2.2, P3 = *Pseudomonas* RSB5.11, P4 = cocktail.

Supplementary Tables

Table S1. Estimated marginal means pairwise comparisons of anti-*Bd* bacterial ASV richness. Contrasts are on a log_{10} scale and degrees of freedom are given from the Kenward-Roger method. P-values were adjusted for multiple comparisons using Tukey's method. Treatments are coded as follows: C = no-probiotic control, P1 = *Pseudomonas* RSB5.4, P2 = *Stenotrophomonas* THA2.2, P3 = *Pseudomonas* RSB5.11, P4 = cocktail.

**Comparison**	**Estimate**	**SE**	**df**	**t-ratio**	**P-value**
C - P1	-0.0383	0.0476	120	-0.804	0.929
C - P2	0.0809	0.0467	120	1.733	0.418
C - P3	0.0662	0.0463	120	1.432	0.609
C - P4	-0.0888	0.0463	120	-1.920	0.312
P1 - P2	0.119	0.0480	121	2.481	0.102
P1 - P3	0.1045	0.0476	120	2.195	0.189
P1 - P4	-0.0506	0.0476	120	-1.063	0.825
P2 - P3	-0.0147	0.0467	120	-0.315	0.998
P2 - P4	-0.1698	0.0467	120	-3.636	*0.0037*
P3 - P4	-0.1551	0.0463	120	-3.352	*0.0093*

Table S2. Estimated marginal means pairwise comparisons of anti-*Bd* bacterial ASV richness with the corresponding probiotics removed (subtracting 0–3 for each individual frog and time point depending on presence/absence of P1, P2, and P3). Contrasts are on a log_{10} scale and degrees of freedom are given from the Kenward-Roger method. P-values were 4adjusted for multiple comparisons using Tukey's method. Treatments are coded as follows: C = no-probiotic control, P1 = *Pseudomonas* RSB5.4, P2 = *Stenotrophomonas* THA2.2, P3 = *Pseudomonas* RSB5.11, P4 = cocktail.

**Comparison**	**Estimate**	**SE**	**df**	**t-ratio**	**P-value**
C - P1	0.0918	0.0656	121	1.400	0.6288
C - P2	0.1906	0.0644	121	2.962	*0.0296*
C - P3	0.2357	0.0638	121	3.697	*0.0030*
C - P4	0.1578	0.0638	121	2.475	0.1032
P1 - P2	0.0988	0.0662	122	1.493	0.5693
P1 - P3	0.1439	0.0656	121	2.194	0.1891
P1 - P4	0.0660	0.0656	121	1.006	0.8524
P2 - P3	0.0451	0.0644	121	0.701	0.9559
P2 - P4	-0.0328	0.0644	121	-0.510	0.9862
P3 - P4	-0.0779	0.0638	121	-1.222	0.7384

Table S3. Estimated marginal means pairwise comparisons of anti-*Bd* bacterial relative abundance. Contrasts are on a $\log_{10}$ scale and degrees of freedom are given from the Kenward-Roger method. P-values were adjusted for multiple comparisons using Tukey's method. Treatments are coded as follows: C = no-probiotic control, P1 = *Pseudomonas* RSB5.4, P2 = *Stenotrophomonas* THA2.2, P3 = *Pseudomonas* RSB5.11, P4 = cocktail.

**Comparison**	**Estimate**	**SE**	**df**	**t-ratio**	**P-value**
C - P1	0.02344	0.00669	124	3.504	*0.0057*
C - P2	0.03540	0.00656	123	5.395	*<0.0001*
C - P3	0.03203	0.00650	123	4.924	*<0.0001*
C - P4	0.03099	0.00650	123	4.765	*0.0001*
P1 - P2	0.01196	0.00674	125	1.773	0.3936
P1 - P3	0.00859	0.00669	124	1.285	0.7011
P1 - P4	0.00755	0.00669	124	1.129	0.7907
P2 - P3	-0.00337	0.00656	123	-0.513	0.9859
P2 - P4	-0.00441	0.00656	123	-0.672	0.9622
P3 - P4	-0.00104	0.00650	123	-0.160	0.9999

Table S4. Result of permutational multivariate analysis of variance (PERMANOVA) using Bray-Curtis dissimilarity matrices. Results from day 0, week 1, week 2, and week 3 are given, respectively.

**Time**	**df (treatment, residual, total)**	**Sum of Squares (treatment, residual, total)**	**R^2 (treatment, residual, total)**	**F**	**P**
Day 0	4, 75, 79	1.20, 8.75, 9.94	0.12, 0.88, 1.00	2.56	≤0.001
Week 1	4, 82, 86	2.79, 12.68, 15.47	0.18, 0.82, 1.00	4.50	≤0.001
Week 2	4, 24, 28	1.20, 4.42, 5.62	0.21, 0.79, 1.00	1.62	0.009
Week 3	4, 25, 29	1.15, 6.18, 7.33	0.16, 0.84, 1.00	1.17	0.154

Table S5. Summary statistics of pairwise treatment comparisons. Results are derived from Bray-Curtis dissimilarity matrices. Treatments are coded as follows: C = no-probiotic control, P1 = *Pseudomonas* RSB5.4, P2 = *Stenotrophomonas* THA2.2, P3 = *Pseudomonas* RSB5.11, P4 = cocktail. Timepoints are coded as follows: D0 = day 0, W1 = week 1, W2 = week 2, and W3 = week 3.

Comparison	df	Sum of Squares	F (model)	R^2	P-value	P-value (adjusted)
CD0 vs P1D0	1	0.47	3.42	0.10	0.016	0.16
CD0 vs P2D0	1	0.43	2.97	0.093	0.026	0.26
CD0 vs P3D0	1	0.50	3.52	0.11	0.012	0.12
CD0 vs P4D0	1	0.41	2.93	0.086	0.036	0.36
P1D0 vs P2D0	1	0.14	1.42	0.047	0.201	1.00
P1D0 vs P3D0	1	0.33	3.37	0.10	0.013	0.13
P1D0 vs P4D0	1	0.18	1.87	0.057	0.085	0.85
P2D0 vs P3D0	1	0.20	1.93	0.063	0.070	0.70
P2D0 vs P4D0	1	0.20	1.97	0.062	0.053	0.53
P3D0 vs P4D0	1	0.13	1.24	0.038	0.241	1.00
CW1 vs P1W1	1	0.79	5.07	0.14	≤0.001	≤0.01
CW1 vs P2W1	1	0.74	4.84	0.13	≤0.001	≤0.01
CW1 vs P3W1	1	0.75	4.40	0.11	≤0.001	≤0.01
CW1 vs P4W1	1	0.83	5.01	0.13	≤0.001	≤0.01
P1W1 vs P2W1	1	0.69	5.07	0.14	≤0.001	≤0.01
P1W1 vs P3W1	1	0.90	5.90	0.16	≤0.001	≤0.01
P1W1 vs P4W1	1	0.78	5.21	0.14	≤0.001	≤0.01
P2W1 vs P3W1	1	0.70	4.63	0.12	0.002	0.02
P2W1 vs P4W1	1	0.30	2.01	0.057	0.071	0.071
P3W1 vs P4W1	1	0.50	3.08	0.083	0.015	0.15
CW2 vs P1W2	1	0.38	1.69	0.16	0.100	1.00
CW2 vs P2W2	1	0.40	2.14	0.18	0.030	0.30
CW2 vs P3W2	1	0.32	1.57	0.14	0.115	1.00
CW2 vs P4W2	1	0.40	1.77	0.15	0.070	0.70
P1W2 vs P2W2	1	0.34	2.22	0.20	0.032	0.32
P1W2 vs P3W2	1	0.36	2.07	0.19	0.054	0.54
P1W2 vs P4W2	1	0.23	1.17	0.12	0.262	1.00
P2W2 vs P3W2	1	0.21	1.51	0.13	0.065	0.65
P2W2 vs P4W2	1	0.18	1.16	0.10	0.330	1.00
P3W2 vs P4W2	1	0.17	0.97	0.089	0.452	1.00

Table S6. Result of permutational multivariate analysis of variance (PERMANOVA) using Jaccard dissimilarity matrices. Results from day 0, week 1, week 2, and week 3 are given, respectively.

Time	df (treatment, residual, total)	Sum of Squares (treatment, residual, total)	R^2 (treatment, residual, total)	F	P
Day 0	4, 75, 79	1.87, 23.50, 25.37	0.074, 0.93, 1.00	1.49	≤0.001
Week 1	4, 82, 86	2.52, 25.58, 28.10	0.090, 0.91, 1.00	2.02	≤0.001
Week 2	4, 24, 28	1.80, 7.89, 9.69	0.19, 0.81, 1.00	1.37	≤0.001
Week 3	4, 25, 29	1.76, 8.53, 10.28	0.17, 0.83, 1.00	1.29	≤0.001

Table S7. Summary statistics of pairwise treatment comparisons. Results are derived from Jaccard dissimilarity matrices. Treatments are coded as follows: C = no-probiotic control, P1 = *Pseudomonas* RSB5.4, P2 = *Stenotrophomonas* THA2.2, P3 = *Pseudomonas* RSB5.11, P4 = cocktail. Timepoints are coded as follows: D0 = day 0, W1 = week 1, W2 = week 2, and W3 = week 3.

**Comparison**	**df**	**Sum of Squares**	**F (model)**	**R^2**	**P-value**	**P-value (adjusted)**
CD0 vs P1D0	1	0.70	2.28	0.071	≤0.001	≤0.01
CD0 vs P2D0	1	0.56	1.71	0.056	0.005	0.05
CD0 vs P3D0	1	0.43	1.29	0.041	0.054	0.54
CD0 vs P4D0	1	0.49	1.52	0.047	0.023	0.23
P1D0 vs P2D0	1	0.34	1.14	0.038	0.126	1.00
P1D0 vs P3D0	1	0.45	1.49	0.047	0.002	0.02
P1D0 vs P4D0	1	0.48	1.64	0.050	0.001	0.01
P2D0 vs P3D0	1	0.36	1.12	0.037	0.161	1.00
P2D0 vs P4D0	1	0.49	1.58	0.050	0.003	0.03
P3D0 vs P4D0	1	0.38	1.20	0.037	0.089	0.89
CW1 vs P1W1	1	0.68	2.16	0.063	≤0.001	≤0.01
CW1 vs P2W1	1	0.70	2.26	0.064	≤0.001	≤0.01
CW1 vs P3W1	1	0.71	2.23	0.062	≤0.001	≤0.01
CW1 vs P4W1	1	0.66	2.07	0.057	≤0.001	≤0.01
P1W1 vs P2W1	1	0.58	1.92	0.058	≤0.001	≤0.01
P1W1 vs P3W1	1	0.67	2.16	0.063	≤0.001	≤0.01
P1W1 vs P4W1	1	0.61	1.94	0.057	≤0.001	≤0.01
P2W1 vs P3W1	1	0.51	1.66	0.048	≤0.001	≤0.01
P2W1 vs P4W1	1	0.63	2.03	0.058	≤0.001	≤0.01
P3W1 vs P4W1	1	0.56	1.78	0.050	≤0.001	≤0.01
CW2 vs P1W2	1	0.44	1.25	0.12	0.028	0.28
CW2 vs P2W2	1	0.48	1.46	0.13	0.010	0.10
CW2 vs P3W2	1	0.50	1.47	0.13	0.011	0.11
CW2 vs P4W2	1	0.55	1.57	0.14	0.002	0.02
P1W2 vs P2W2	1	0.40	1.27	0.12	0.007	0.07
P1W2 vs P3W2	1	0.46	1.39	0.13	0.007	0.07
P1W2 vs P4W2	1	0.39	1.14	0.11	0.099	0.99
P2W2 vs P3W2	1	0.41	1.35	0.12	0.002	0.02
P2W2 vs P4W2	1	0.46	1.47	0.13	0.009	0.09
P3W2 vs P4W2	1	0.41	1.25	0.11	0.024	0.24
CW3 vs P1W3	1	0.50	1.44	0.13	0.010	0.10
CW3 vs P2W3	1	0.52	1.60	0.14	0.004	0.04
CW3 vs P3W3	1	0.45	1.28	0.11	0.028	0.28
CW3 vs P4W3	1	0.46	1.33	0.12	0.004	0.04
P1W3 vs P2W3	1	0.46	1.41	0.12	0.005	0.05
P1W3 vs P3W3	1	0.42	1.20	0.11	0.037	0.37
P1W3 vs P4W3	1	0.42	1.20	0.11	0.039	0.39
P2W3 vs P3W3	1	0.38	1.14	0.10	0.068	0.68
P2W3 vs P4W3	1	0.42	1.27	0.11	0.009	0.09
P3W3 vs P4W3	1	0.36	1.02	0.093	0.372	1.00

Table S8. Correlation matrix of immunity gene expression using Pearson's correlation coefficient.

	CSF1	FOXP3	IL10	IL34	TGFB	TLR2	TLR6	TNFA
**CSF1**	1.0000000	0.2992808	-0.3476719	-0.3680482	-0.3677570	-0.2865985	-0.2916561	-0.3482680
**FOXP3**	0.2992808	1.0000000	0.1382658	0.1319526	0.1329778	0.1738916	0.1908799	0.1423729
**IL10**	-0.3476719	0.1382658	1.0000000	0.9892532	0.9891197	0.9249106	0.9797125	0.9842874
**IL34**	-0.3680482	0.1319526	0.9892532	1.0000000	0.9999704	0.8976541	0.9656938	0.9718451
**TGFB**	-0.3677570	0.1329778	0.9891197	0.9999704	1.0000000	0.8975934	0.9655103	0.9717494
**TLR2**	-0.2865985	0.1738916	0.9249106	0.8976541	0.8975934	1.0000000	0.8864516	0.9195672
**TLR6**	-0.2916561	0.1908799	0.9797125	0.9656938	0.9655103	0.8864516	1.0000000	0.9472430
**TNFA**	-0.3482680	0.1423729	0.9842874	0.9718451	0.9717494	0.9195672	0.9472430	1.0000000

Table S9. Pairwise comparisons of *FOXP3* expression across treatments. Comparisons are completed using Dunn's test. The Benjamini-Hochberg method was used for p-value adjustment of multiple comparisons. Treatments are coded as follows: C = no-probiotic control, P1 = *Pseudomonas* RSB5.4, P2 = *Stenotrophomonas* THA2.2, P3 = *Pseudomonas* RSB5.11, P4 = cocktail.

Comparison	Z	P-value (unadjusted)	P-value (adjusted)
C - P1	2.981	0.00288	0.0288
C - P2	1.457	0.145	0.290
P1 - P2	-1.339	0.180	0.301
C - P3	2.0723	0.0382	0.127
P1 - P3	-0.693	0.488	0.611
P2 - P3	0.596	0.551	0.551
C - P4	0.596	0.551	0.612
P1 - P4	-2.602	0.00927	0.0463
P2 - P4	-0.986	0.324	0.463
P3 - P4	-1.645	0.0999	0.250

Table S10. Pairwise comparisons of *TNFA* expression across treatments. Comparisons are completed using Dunn's test. The Benjamini-Hochberg method was used for p-value adjustment of multiple comparisons. Treatments are coded as follows: C = no-probiotic control, P1 = *Pseudomonas* RSB5.4, P2 = *Stenotrophomonas* THA2.2, P3 = *Pseudomonas* RSB5.11, P4 = cocktail.

Comparison	Z	P-value (unadjusted)	P-value (adjusted)
C - P1	-0.345	0.730	0.730
C - P2	-1.118	0.264	0.439
P1 - P2	-0.781	0.435	0.544
C – P3	0.642	0.521	0.579
P1 – P3	0.986	0.324	0.463
P2 - P3	1.746	0.0809	0.202
C – P4	2.270	0.0232	0.0774
P1 – P4	2.615	0.00893	0.0447
P2 – P4	3.338	0.000844	0.00844
P3 – P4	1.628	0.103	0.207
